# Changes in hepatic fibrosis and vitamin D levels after viral hepatitis C eradication using direct-acting antiviral therapy

**DOI:** 10.1186/s12876-020-01485-8

**Published:** 2020-10-17

**Authors:** Supachaya Sriphoosanaphan, Kessarin Thanapirom, Sirinporn Suksawatamnuay, Panarat Thaimai, Sukanya Sittisomwong, Kanokwan Sonsiri, Nunthiya Srisoonthorn, Nicha Teeratorn, Nattaporn Tanpowpong, Bundit Chaopathomkul, Sombat Treeprasertsuk, Yong Poovorawan, Piyawat Komolmit

**Affiliations:** 1 Division of Gastroenterology, Department of Medicine, Faculty of Medicine, Chulalongkorn University and King Chulalongkorn Memorial Hospital, Bangkok, Thailand; 2 Center of Excellence in Liver Diseases, King Chulalongkorn Memorial Hospital, Thai Red Cross, Bangkok, Thailand; 3 Liver Fibrosis and Cirrhosis Research Unit, Chulalongkorn University, Bangkok, Thailand; 4 Department of Radiology, Faculty of Medicine, Chulalongkorn University and King Chulalongkorn Memorial Hospital, Bangkok, Thailand; 5 Center of Excellence in Clinical Virology, Faculty of Medicine, Chulalongkorn University, Bangkok, Thailand

**Keywords:** Vitamin D, Hepatitis C, Liver fibrosis, Direct-acting antiviral, Amino terminal of type III procollagen peptide

## Abstract

**Background:**

Vitamin D (VD) is important in hepatic fibrogenesis in animal models and human studies. VD deficiency is associated with liver fibrosis progression. Metabolic dysfunction of the liver, as an intermediate organ for VD metabolism, contributes partly to this deficiency. We hypothesized that improving hepatic fibrosis and inflammation in chronic hepatitis C (CHC) patients after eradication with direct-acting antivirals (DAA) would increase 25-hydroxyVD [25(OH)VD] levels.

**Methods:**

Eighty CHC patients (17 chronic hepatitis, and 63 cirrhosis) were enrolled. Baseline characteristics, hepatitis C viral load (VL), genotypes, liver enzymes and liver stiffness measurements (LSM) were assessed at baseline. Blood samples for 25(OH)VD and the procollagen type III N-terminal peptide (P3NP) were collected at baseline, 24 and 48 weeks. LSMs were re-evaluated at 48 weeks. Serum 25(OH)VD levels < 30 ng/mL were defined as VD insufficiency/deficiency. Paired *t*-tests were used for statistical analyses.

**Results:**

Among 80 patients, the mean age was 57.7 ± 10.5 years, and 52.5% were men. The mean VL was 6.1 ± 0.7 logIU/mL with genotype 1 predominance (55%). All patients achieved sustained virological response. The alanine aminotransferase levels decreased from 79.9 ± 53.3 U/L at baseline to 25.7 ± 17.2 and 22.3 ± 11.0 U/L at 24 and 48 weeks, respectively (*p* < 0.001). The mean LSM decreased from 19.2 ± 15.3 to 11.7 ± 8.0 kPa at 48 weeks (*p* < 0.001). The P3NP levels decreased from 43.6 ± 22.0 ng/mL before treatment to 35.7 ± 21.1 and 29.4 ± 15.0 ng/mL at 24 and 48 weeks, respectively (*p* < 0.001). The proportions of VD insufficiency/deficiency were 72.5%, 91.3%, and 86.5% at baseline, 24 and 48 weeks, respectively. The 25(OH)VD levels decreased from 26.3 ± 10.7 ng/mL at baseline to 20.8 ± 8.1 and 20.8 ± 8.5 ng/mL at 24 and 48 weeks, respectively (*p* < 0.001).

**Conclusions:**

Curative treatment with DAA attenuated the liver stiffness and inflammation but did not improve VD levels. Over 80% of patients remained VD insufficient/deficient. Whether VD replacement during and after DAA therapy can improve hepatic fibrosis remains unclear.

*Trial registration* The Thai Clinical Trial Registry as TCTR20161025001 (31 October 2016). http://www.clinicaltrials.in.th/index.php?tp=regtrials&menu=trialsearch&smenu=fulltext&task=search&task2=view1&id=2136.

## Background

Vitamin D (VD) deficiency is the most common nutritional deficiency and affects millions of people worldwide [1]. Apart from insufficient sunlight exposure, chronic liver disease and genetic variances in the genes involved in VD metabolism are the major causes of VD deficiency [2, 3]. VD is primarily involved in calcium homeostasis and plays important roles in the immune system, cell differentiation and proliferation, and hepatic fibrogenesis [4, 5]. The liver is an intermediate organ in VD metabolism that hydroxylates preVD to 25-hydroxyVD [25(OH)VD], and serum levels of 25(OH)VD represent the VD status. Levels between 20 and 30 and < 20 ng/mL indicate VD insufficiency and deficiency, respectively [6].

Chronic liver diseases are one cause of VD deficiency, and the degree depends on the disease severity [7]. One proposed explanation for this deficiency is decreased liver metabolic functions due to hepatic injury and fibrosis [3]. Apart from affecting general health, VD deficiency promotes and exacerbates fibrogenic processes in addition to the liver injury caused by the primary etiology of chronic liver diseases such as hepatitis C virus (HCV). Eradicating the cause of chronic liver diseases attenuates hepatic inflammation, the degree of hepatic fibrosis, and liver disease severity as assessed by the Child-Turcotte-Pugh (CTP) and model for end-stage liver disease (MELD) scores [8, 9]. These scoring systems are based partly on or reflect synthetic or metabolic liver functions such as albumin and coagulation factors.

HCV is a major cause of chronic liver disease, and its complications affect millions of people worldwide. The recent development of a new generation of direct-acting antivirals (DAA) has resulted in a > 90% cure rate [10]. HCV eradication has been shown to improve hepatic inflammation and functions within a few months [11], as well as improve hepatic fibrosis after cure [12, 13].

VD deficiency is common among patients with chronic hepatitis C (CHC), with a prevalence near 90% [7, 14]. To our knowledge, no data exist that show how HCV eradication affects hepatic VD metabolic functions. We hypothesized that attenuated hepatic inflammation and fibrosis due to curing HCV with DAA may positively affect VD synthesis, resulting in increased VD levels.

## Methods

### Patients and study design

This retrospective cohort study was part of a previous project evaluating CHC patients who underwent DAA treatment between November 2016 and December 2017. The study was conducted at the outpatient department of King Chulalongkorn Memorial Hospital, a tertiary referral center and academic teaching hospital in Bangkok, Thailand. Blood samples and medical records were retrieved from 80 CHC patients. We analyzed the available data from blood samples collected for other research purposes and liver stiffness measurements (LSM), which were performed as a standard of care.

The inclusion criteria were naïve or nonresponder CHC patients and age between 18 and 80 years old. The exclusion criteria were history of VD supplements within the last 3 months, decompensated liver cirrhosis, coinfection with hepatitis B virus (HBV) or human immunodeficiency virus, autoimmune diseases, history of steroid or immunosuppressive therapy, or history of interferon treatment within 12 months.

**Fig. 1:**
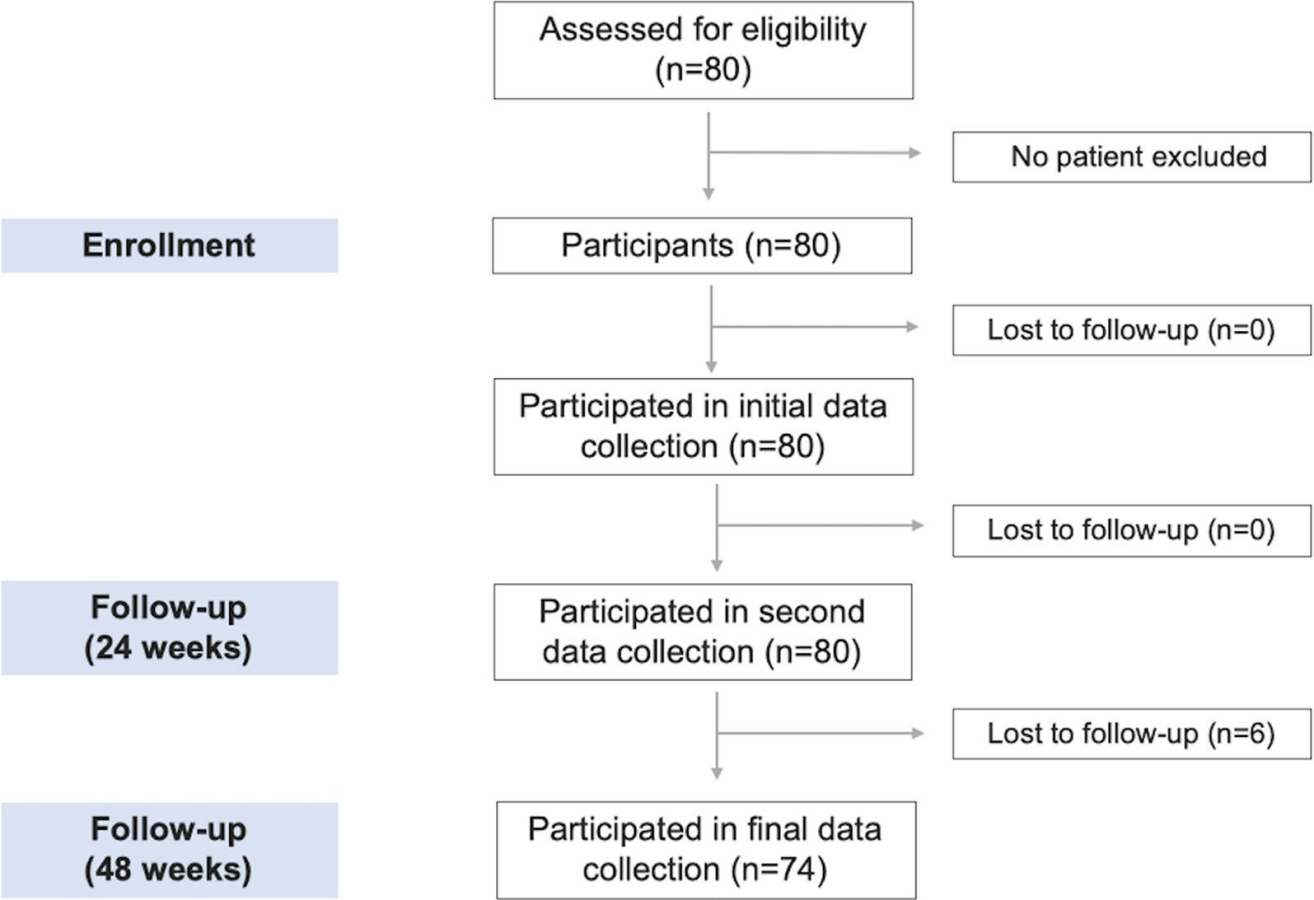
Flow diagram of the study

All CHC patients completed a DAA regimen according to their HCV genotype and liver fibrosis stage. After initiating treatment, patients attended regular follow-up visits, and their serum transaminase levels were monitored at each appointment. Blood samples were collected at baseline, 24 and 48 weeks after enrollment. Baseline characteristics, HCV viral load (VL), HCV genotypes, liver enzymes, procollagen type III N-terminal peptide (P3NP), and serum 25(OH)VD levels were assessed. LSM was performed using Fibroscan® (Echosens, Paris, France) at baseline and reevaluated at 48 weeks. Blood samples were stored at − 70 °C and simultaneously assessed for 25(OH)VD and P3NP levels at the end of the study. Figure 1 shows the study protocol.

### Laboratory methods

#### Measurement of VD levels

Serum 25(OH)VD levels were measured using the Liaison 25(OH)VD total assay (DiaSorin, Saluggia, Italy), which was performed on the LIAISON® chemiluminescence analyzer following the manufacturer’s instructions. Final concentrations are reported in ng/mL.

According to the Endocrine Society Practice Guidelines [15], the criteria for VD insufficiency/deficiency was defined as serum 25(OH)VD < 30 ng/mL. Serum 25(OH)VD concentrations < 20 ng/mL and between 20 and 29 ng/mL were defined as VD deficiency and VD insufficiency, respectively.

#### Measurement of P3NP levels

Serum concentrations of P3NP were measured via the quantitative sandwich enzyme-linked immunosorbent assay technique following the manufacturer’s instructions (Cloud-Clone Corp., Houston, TX, USA). The results were calculated by referencing the standard curve.

### Statistical analysis

Baseline characteristics are presented as the percentage or mean ± standard deviation (SD). Categorical variables were analyzed using chi-square or Fisher’s exact tests when appropriate. Continuous variables with a normal distribution were compared within groups (pre- and post-treatment) with dependent *t*-tests; variables with a skewed distribution were compared using the Wilcoxon signed-rank test. The normality of continuous data was assessed by visually inspecting histograms, and conducting a Shapiro-Wilks test. All statistical analyses were performed in SPSS Version 22.0 (IBM Corp., Armonk, NY, USA). Differences were considered significant at *p* < 0.05.

### Ethical approval and consent to participate

The Ethics Committee of the Institutional Review Board at Chulalongkorn University, Bangkok, Thailand, reviewed and approved the study in accordance with the Declaration of Helsinki (1989) of the World Medical Association (IRB No.: 424/59). The study was registered with the Thai Clinical Trial Registry as TCTR20161025001 on 31 October 2016 (Additional files 1 and 2: Supplementary data 1 and 2). All patients enrolled in this study provided written informed consent and consent for publication.

## Results

### General characteristics

From November 2016 through December 2017, 80 CHC patients completed DAA treatment. Table 1 summarizes their baseline demographic characteristics and laboratory data.

**Table 1:** Baseline characteristics of CHC patients

Characteristics	Total population (n = 80)
Age (years)	57.7 ± 10.5
Sex: male, n (%)	42 (52.5)
Body mass index (kg/m^2^)	24.4 ± 3.5
Naïve cases, n (%)	42 (52.5)
Cirrhosis, n (%)	63 (78.8)
Child–Pugh class A	58 (72.5)
Child–Pugh class B	5 (6.3)
Liver stiffness (kPa)^a^	19.2 ± 15.3
HCV viral load (log IU/mL)	6.1 ± 0.7
Genotype, n (%)	
1	44 (55.0)
3	30 (37.5)
6	6 (7.5)
Alanine aminotransferase level (U/L)	80.0 ± 53.3
Treatment regimen, n (%)^b^	
SOF/DAC	22 (27.5)
SOF/LED	8 (10.0)
SOF/VEL	1 (1.25)
SOF/DAC/RBV	42 (52.5)
SOF/LED/RBV	6 (7.5)
SOF/VEL/RBV	1 (1.25)
Sustained virological response, n (%)	80 (100)

^a^n = 49

^b^DAC, daclatasvir; LED, ledipasvir; RBV, ribavirin; SOF, sofosbuvir; VEL, velpatasvir

Of the patients, 52.5% were male with a mean age of 57.7 years (range 33–77 years); 63 (78.8%) had compensated liver cirrhosis; 58 (72.5%) were Child–Pugh class A, and 5 (6.3%) were Child–Pugh class B. Liver cirrhosis was diagnosed according to clinical basis, including laboratory tests or LSM ≥ 12.5 kPa [16] or imaging consistent with cirrhosis. Most patients had HCV genotype 1 (n = 44, 55.0%) with a mean log HCV VL of 6.1 logIU/mL. More than half the patients (n = 42, 52.5%) were naïve (Additional file 3: Supplementary data 3). The DAA treatment regimen consisted of sofosbuvir (SOF), daclatasvir (DAC), ledipasvir (LED), and velpatasvir (VEL). Most patients (n = 42, 52.5%) had been treated with SOF/DAC/ribavirin (RBV). DAA duration depended on fibrosis stage and treatment regimen. At 24 weeks, 59 CHC patients who underwent a 12-week regimen achieved sustained virological response (SVR), whereas the remaining 21 CHC patients completed their 24-week DAA courses, and all achieved SVR within 12 weeks later. Six CHC patients were lost to follow-up at 48 weeks.

### Changes in alanine transaminase (ALT) levels

**Fig. 2:**
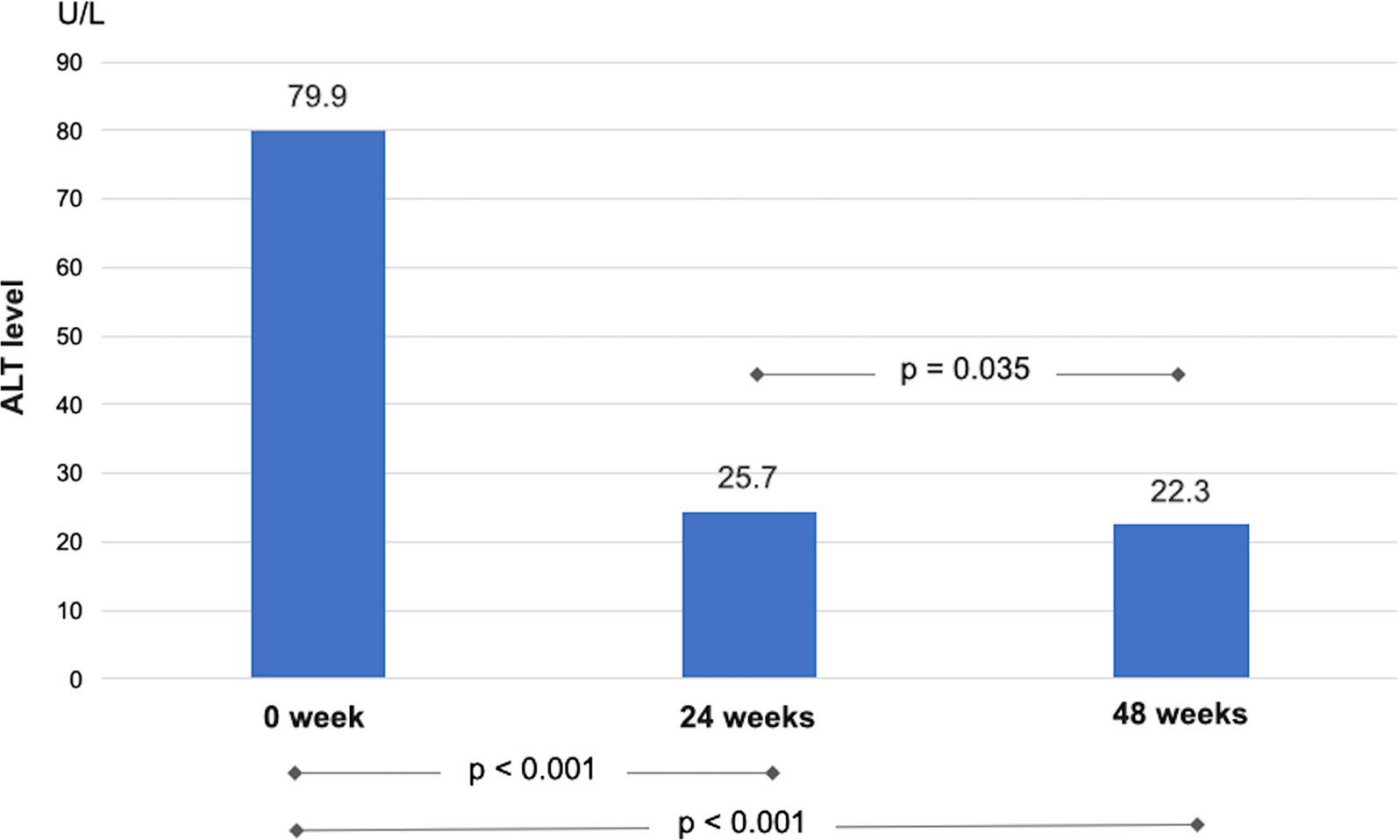
ALT levels in CHC patients at baseline and after DAA curative treatment

Pre-and post-treatment blood samples were taken from 80 CHC patients. After DAA treatment, mean ALT levels decreased significantly from 79.9 ± 53.3 U/L at baseline to 25.7 ± 17.2 U/L and 22.3 ± 11.0 U/L at 24 and 48 weeks, respectively (*p* < 0.001, Fig. 2).

At 24 weeks, 11 (13.8%) CHC patients still had ALT levels > 40 U/L (range 42–119 U/L). ALT levels normalized in all but 3 (3.8%) CHC patients at 48 weeks. Of these three patients with persistent hepatitis (range 42–66 U/L), two were diagnosed with drug-induced liver injury (herb), and one had nonalcoholic steatohepatitis.

### Changes in liver stiffness

**Fig. 3:**
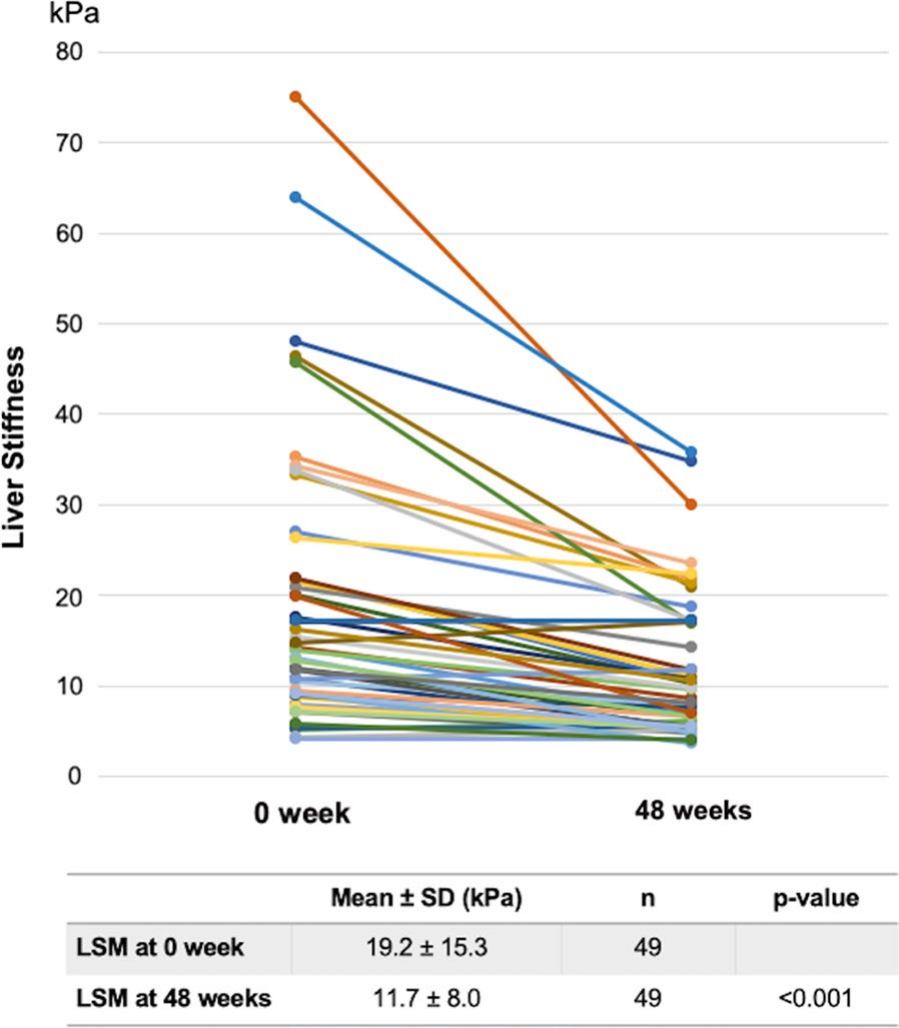
LSM in CHC patients at baseline and after DAA curative treatment

Available paired LSM pre- and post-treatment data were retrieved from 49 of 80 patients. The mean LSM at baseline was 19.2 ± 15.3 kPa, which significantly decreased to 11.7 ± 8.0 kPa at 48 weeks (*p* < 0.001, Fig. 3).

Liver stiffness was greatly reduced after DAA treatment in cirrhotic CHC patients (21.7 ± 15.4 vs. 13.0 ± 8.2 kPa at weeks 0 and 48, respectively; *p* < 0.001) compared with CHC patients without cirrhosis. No significant change in LSM was observed in the non-cirrhotic group (Table 2).

**Table 2:** Changes in liver stiffness between cirrhotic and non-cirrhotic patients

	Week 0 (kPa)	Week 48 (kPa)	*p* value
Cirrhotic (n = 41)	21.7 ± 15.4	13.0 ± 8.2	< 0.001
Non-cirrhotic (n = 8)	5.9 ± 1.4	5.1 ± 0.7	0.141

### Changes in P3NP levels

**Fig. 4:**
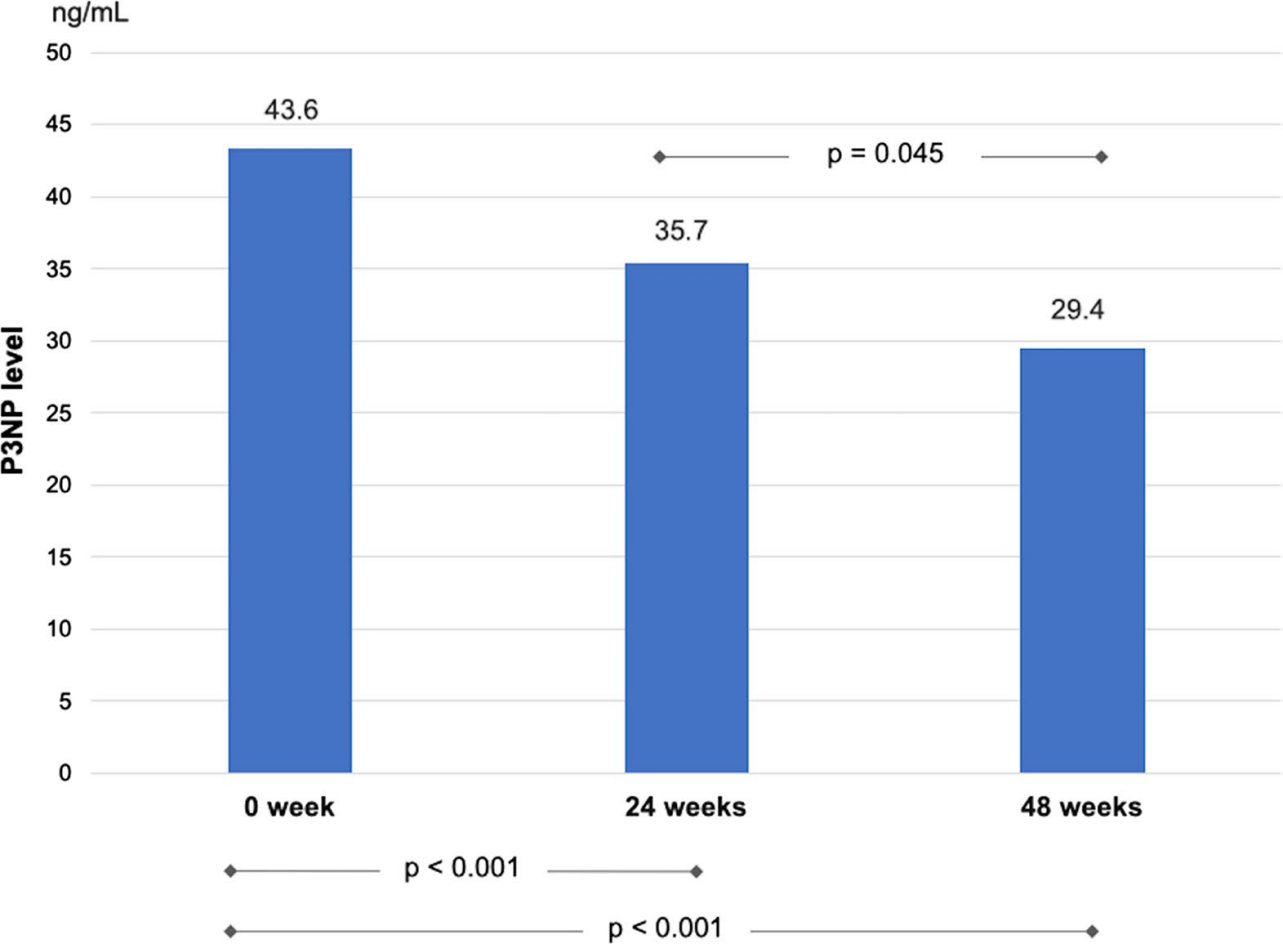
P3NP levels in CHC patients at baseline and after DAA curative treatment

Changes in serum P3NP levels were analyzed from paired blood samples from 80 patients at 24 weeks and 74 patients at 48 weeks. The mean P3NP level was 43.6 ± 22.0 ng/mL before DAA treatment and significantly decreased to 35.7 ± 21.1 and 29.4 ± 15.0 ng/mL at 24 and 48 weeks, respectively (*p* < 0.001, Fig. 4).

### Changes in serum 25(OH)VD levels

**Fig. 5.**
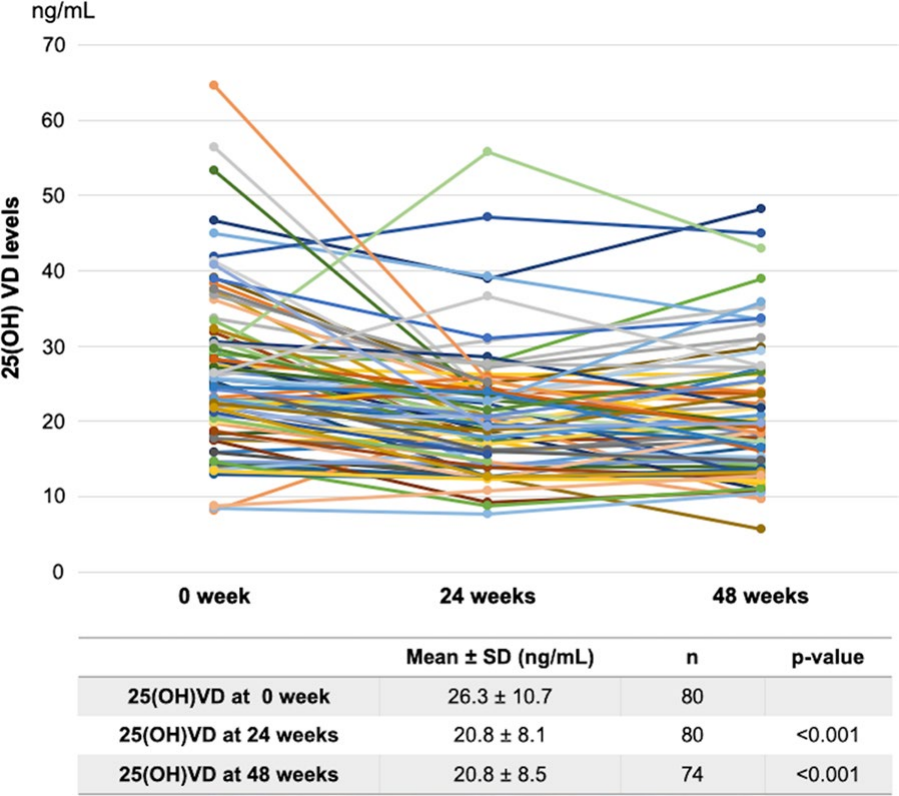
25(OH)VD levels in CHC patients at baseline and after DAA curative treatment

Changes in serum 25(OH)VD levels were assessed from paired blood samples from 80 and 74 patients taken at 24 and 48 weeks, respectively. Serum 25(OH)VD levels decreased significantly from a mean of 26.3 ± 10.7 ng/mL before DAA treatment to 20.8 ± 8.1 and 20.8 ± 8.5 ng/mL at 24 and 48 weeks, respectively (*p* < 0.001, Fig. 5).

Patients with cirrhosis had significantly lower pretreatment 25(OH)VD levels compared with those without cirrhosis (25.0 ± 10.5 vs. 31.0 ± 10.3 ng/mL, *p* = 0.039). Even after curative treatment, cirrhotic patients still had lower 25(OH)VD levels at 48 weeks than non-cirrhotic patients; however, this difference was not statistically significant (19.9 ± 8.2 vs. 24.1 ± 9.4 ng/mL, *p* = 0.087; Table 3).

**Table 3:** Serum 25(OH)VD levels in CHC patients at baseline and after DAA curative treatment comparing cirrhotic and non-cirrhotic patients

	Week 0 (ng/mL)	Week 24 (ng/mL)	Week 48^a^ (ng/mL)
Cirrhotic (n = 63)	25.0 ± 10.5	20.2 ± 8.1	19.9 ± 8.2
Non-cirrhotic (n = 17)	31.0 ± 10.3	22.8 ± 7.7	24.1 ± 9.4
*p* value	0.039	0.251	0.087

^a^Six patients were lost to follow-up at 48 weeks; 5 in cirrhotic group and 1 in non-cirrhotic group

**Fig. 6:**
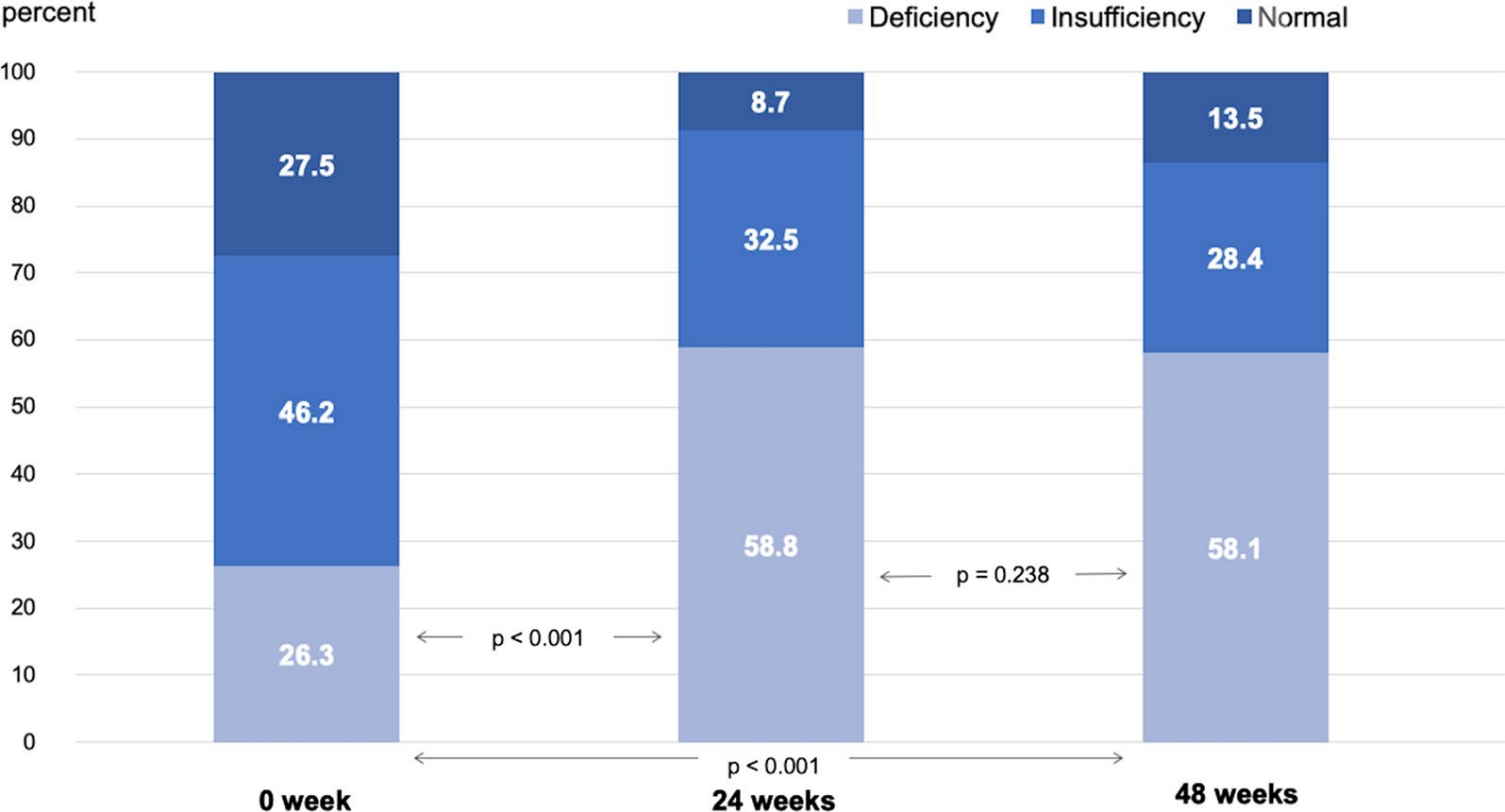
Serum 25(OH)VD level distribution in CHC patients at baseline, 24 weeks, and 48 weeks

The proportions of VD insufficiency/deficiency in CHC patients were 72.5%, 91.3%, and 86.5% at baseline, 24 weeks, and 48 weeks, respectively. Serum 25(OH)VD levels were stratified into three categories: deficiency (< 20 ng/mL), insufficiency (20–29.9 ng/mL), and normal (> 30 ng/mL). Notably, the proportion of patients with VD deficiency significantly increased from 26.3% at baseline to 58.8% and 58.1% at 24 and 48 weeks, respectively (*p* < 0.001, Fig. 6).

### Advanced liver fibrosis

A subgroup analysis was performed based on LSM. Patients with liver stiffness ≥ 9.5 kPa were classified as having advanced liver fibrosis (≥ F3) in accordance with international cutoff guidelines [17, 18]. CHC patients with advanced liver fibrosis had significantly higher ALT and P3NP levels at baseline than those without advanced fibrosis (Table 4). Additionally, pretreatment VD levels were significantly lower in CHC patients with advanced liver fibrosis (21.9 ± 6.4 vs. 26.8 ± 9.1 ng/mL, *p* = 0.036). After DAA treatment, ALT and P3NP levels remained higher at 48 weeks in patients with advanced liver fibrosis. However, VD levels did not significantly differ between groups at 48 weeks.

**Table 4:** Comparison of laboratory data between CHC patients with and without advanced liver fibrosis

Liver stiffness measurement	< F3 (n = 15)	≥ F3 (n = 34)	*p* value
ALT levels (U/L)			
Week 0	56.7 ± 48.0	97.3 ± 60.9	0.027
Week 24	15.4 ± 9.0	32.3 ± 21.9	0.006
Week 48	14.3 ± 6.1	24.5 ± 10.0	0.001
P3NP levels (ng/mL)			
Week 0	35.8 ± 12.1	49.0 ± 14.6	0.004
Week 24	29.4 ± 13.4	38.7 ± 18.5	0.088
Week 48	25.9 ± 5.9	39.3 ± 14.7	0.002
25(OH)VD levels (ng/mL)			
Week 0	26.8 ± 9.1	21.9 ± 6.4	0.036
Week 24	19.2 ± 6.7	19.7 ± 5.2	0.816
Week 48	22.3 ± 8.0	19.4 ± 6.5	0.206

## Discussion

This study analyzed changes in VD levels after curative treatment of HCV at 24 and 48 weeks after treatment initiation. All patients were cured of HCV as confirmed by the 12-week SVR. The retrospective data showed an improvement in the degree of hepatic fibrosis as assessed by liver elastography (Fibroscan®) and decreased P3NP levels. Nearly all patients showed normalized transaminase enzyme levels. Paired serum samples from 80 patients demonstrated no significant improvement in VD levels. In addition, at the end of the study, 86.5% and 58.1% of patients had VD levels below 30 ng/mL (VD insufficiency) and 20 ng/mL (VD deficiency), respectively.

VD is an essential hormonal substance involved in normal organ physiology and pathophysiology in several diseases [1]. High proportions of patients with chronic liver diseases have VD insufficiency and deficiency, which worsen along with the hepatic function and cirrhosis severity [7]. As the liver is an intermediate organ involved in VD synthesis and several lines of evidences and hypotheses suggest that decreased hepatic metabolic functions caused by inflammation and fibrosis may be a major cause of low VD levels [3]. This mechanism was the initial hypothesis of our study. Other potential causes include a lack of ultraviolet (UV) exposure, poor intake due to underlying liver diseases (especially patients with decompensated liver diseases), and underlying genetic variations in the genes involved in VD metabolism [3].

After entering hepatocytes, cholecalciferol is metabolized through microsomal enzymes and cytochrome P450 (CYP), mainly CYP2R1, also known as the 25-hydroxylase enzyme [19]. The function of this enzyme can be assessed by measuring transcription levels in hepatic tissues, but this approach is not applicable to the clinical outcome of interest [20]. A large study on various quantitative liver function tests (QLFTs) involving bile acid and dynamic and metabolic functional assessments in CHC patients with advanced fibrosis and cirrhosis showed an add-on benefit in predicting clinical outcomes [21]. However, these QLFTs represent global, not specific, hepatic function. Thus, in our study, we measured VD levels rather than CYP2R1 function to explore the effect of treatment on VD deficiency. Our study showed that upon curing HCV with decreases in hepatic fibrosis and inflammation, 25(OH)VD levels remained lower than 30 ng/mL in nearly 90% of patients. These patients were not asked to change their lifestyles or food intake, and nearly all of them had compensated liver diseases with no reason for limitations in their daily activities. Previous data showed that approximately 39% and 43% of the Thai general population and residents in the central part of Thailand, respectively, had VD levels below 30 ng/mL, and 7% of the latter were VD deficient (< 20 ng/mL) [22]. The high rate of CHC patients with VD deficiency that did not improve along with enhanced hepatic functions was not be clarified by our study setting. Several other factors may have affected the results. Variations in genetic background causing VD deficiency have been shown in several large studies [2, 23], as well as our data in Thai patients with chronic hepatitis B and C [24, 25]. The remaining VD deficiency after DAA therapy might be explained by other uncorrected factors rather than poor metabolic liver function. To put it another way, impaired liver function might not be a major cause contributing to this nutritional deficiency in CHC patients. In addition, although Thailand experiences heavy sunlight and UV-B exposure year-round, many people, especially those in urban areas, prefer to remain in the shade. Further, food type and amount are not normally a major cause of VD deficiency. Except for VD acquired from supplements or fortified milk, foods high in VD that contain 1000–2000 units per day are recommended [6]. However, a study by Lange et al. demonstrates a trend towards a lower incidence of VD deficiency at 24 weeks after treatment completion with interferon [14]. This finding suggests that hepatic VD metabolism, especially 25-alpha hydroxylation, might take time to be restored and show clinical benefit. A relatively short-time period in the current study may not allow observation of a demonstrable VD change. Longer VD level monitoring is required to explore this hypothesis. Nevertheless, the most crucial result of the present study is that VD deficiency persists in CHC patients after being cured of HCV.

The influence of VD on chronic liver diseases has been thoroughly described in various studies regardless of etiologies. Serum VD levels were inversely correlated with HBV VL in patients with chronic hepatitis B [26, 27]. Low serum 25(OH)VD levels were associated with advanced disease and were also a predictor of response with ursodeoxycholic acid in primary biliary cholangitis patients [28]. Additionally, low serum VD levels were related to severe histological features and potentially predicted unfavorable treatment outcomes in patients with autoimmune hepatitis [29]. Among CHC patients, VD deficiency has several detrimental effects, including dysregulation of T-cell functions, resulting in lower response rates of the HCV genotype 1 to pegylated interferon and RBV treatment [14, 30]. However, the revolutionization of HCV treatment with DAA has improved the overall curative rate to nearly 100% [10]. The issues of immune dysregulation and VD replacement to improve the cure rate are negligible. What is left for the researches on VD in liver diseases is its effect on hepatic fibrogenesis, which is immensely important [4, 31]. VD deficiency negatively affects hepatic fibrosis. VD replacement may help prevent and improve hepatic fibrosis. Our recent study on CHC patients showed that short-term VD supplementation can improve serum markers associated with fibrogenesis [32].

Improvement in liver functions and hepatic fibrosis after curative treatment of HCV in the era of DAA has been clearly demonstrated. Both decreased hepatitis and reduced MELD/CTP scores usually occur during the early period of treatment and continuously improve throughout the 12-week course of therapy and after the disease is cured (SVR 12 and 24 weeks) [8, 11]. Nowadays, follow-up assessments of hepatic fibrosis by liver biopsy are increasingly rare and have been replaced by various liver stiffness assessment tools such as transient elastography, ultrasound elastography, and magnetic resonance elastography. Our study demonstrated a significant decrease in liver stiffness scores within 6 and 12 months after starting DAA treatment, as reported by others [33, 34]. However, the elevation in stiffness scores could be falsely increased or masked by the degree of transaminitis [35]. Additionally, the decrease of LSM early after DAA treatment did not truly reflect liver fibrosis regression but rather than a significant reduction in degree of necroinflammation [36]. Therefore, we added a paired study of the P3NP levels as a fibrogenic marker to demonstrate the improved hepatic fibrosis in our patients. This peptide is generated during the process of type 3 collagen synthesis and has been used with moderate accuracy in a follow-up study of hepatic fibrosis [37].

The results should be considered in the context of some limitations. As a retrospective study, patients’ activities and food intake could not be recommended or recorded. However, patients with histories of taking VD supplements were excluded. In addition, this study evaluated only the clinical improvement in hepatic inflammation and fibrosis. No QLFTs were included in the study to assess minor improvements in global hepatic function, as the standard LFTs could not show changes in dynamic metabolic functions. Lastly, the small numbers of participants in the cohort might not provide a strong conclusion. Hence, a larger confirmatory or a population-based design is warranted in a future study.

## Conclusions

In summary, the present study demonstrated improvements in hepatic inflammation and fibrosis within 24 and 48 weeks after starting DAA treatment in CHC patients who achieved SVR. Importantly, over 80% of the CHC patients had VD insufficiency/deficiency before HCV treatment, which did not improve after curative HCV treatment and reduction of hepatic inflammation and fibrosis.

VD is normally required for general health and to prevent several diseases. VD supplementation in patients with chronic liver diseases with persistent hepatic fibrosis is another issue to be explored in view of the benefit of VD in accelerating fibrolytic processes.

## Supplementary information

**Additional file 1:** Supplementary data 1: Study protocol (Original version).

**Additional file 2:** Supplementary data 2: Study protocol (English version).

**Additional file 3:** Supplementary data 3: Data set.

## Data Availability

The datasets used and/or analysed during the current study are available from the corresponding author on reasonable request.

## Abbreviations

CHC: Chronic hepatitis C
DAA: Direct-acting antivirals
HCV: Hepatitis C virus
LSM: Liver stiffness measurement
P3NP: Procollagen type III N-terminal peptide
SVR: Sustained virological response
VD: Vitamin D
VL: Viral load
25(OH)VD: 25-Hydroxyvitamin D

## References

[1] Holick MF (2007). Vitamin D deficiency. N Engl J Med.

[2] Jiang X, O'Reilly PF, Aschard H, Hsu YH, Richards JB, Dupuis J, Ingelsson E, Karasik D, Pilz S, Berry D (2018). Genome-wide association study in 79,366 European-ancestry individuals informs the genetic architecture of 25-hydroxyvitamin D levels. Nat Commun.

[3] Kitson MT, Roberts SK (2012). D-livering the message: the importance of vitamin D status in chronic liver disease. J Hepatol.

[4] Ding N, Yu RT, Subramaniam N, Sherman MH, Wilson C, Rao R, Leblanc M, Coulter S, He M, Scott C (2013). A vitamin D receptor/SMAD genomic circuit gates hepatic fibrotic response. Cell.

[5] Hewison M (2012). An update on vitamin D and human immunity. Clin Endocrinol (Oxf).

[6] Holick MF, Binkley NC, Bischoff-Ferrari HA, Gordon CM, Hanley DA, Heaney RP, Murad MH, Weaver CM, Endocrine S (2011). Evaluation, treatment, and prevention of vitamin D deficiency: an Endocrine Society clinical practice guideline. J Clin Endocrinol Metab.

[7] Petta S, Camma C, Scazzone C, Tripodo C, Di Marco V, Bono A, Cabibi D, Licata G, Porcasi R, Marchesini G (2010). Low vitamin D serum level is related to severe fibrosis and low responsiveness to interferon-based therapy in genotype 1 chronic hepatitis C. Hepatology (Baltimore, MD).

[8] Charlton M, Everson GT, Flamm SL, Kumar P, Landis C, Brown RS, Fried MW, Terrault NA, O'Leary JG, Vargas HE (2015). Ledipasvir and sofosbuvir plus ribavirin for treatment of HCV infection in patients with advanced liver disease. Gastroenterology.

[9] Marcellin P, Gane E, Buti M, Afdhal N, Sievert W, Jacobson IM, Washington MK, Germanidis G, Flaherty JF, Aguilar Schall R (2013). Regression of cirrhosis during treatment with tenofovir disoproxil fumarate for chronic hepatitis B: a 5-year open-label follow-up study. Lancet (London, England).

[10] Pawlotsky J-M, Negro F, Aghemo A, Berenguer M, Dalgard O, Dusheiko G, Marra F, Puoti M, Wedemeyer H (2018). EASL recommendations on treatment of hepatitis C 2018. J Hepatol.

[11] Curry MP, O'Leary JG, Bzowej N, Muir AJ, Korenblat KM, Fenkel JM, Reddy KR, Lawitz E, Flamm SL, Schiano T (2015). Sofosbuvir and velpatasvir for HCV in patients with decompensated cirrhosis. N Engl J Med.

[12] Labarga P, Fernandez-Montero JV, Barreiro P, Pinilla J, Vispo E, de Mendoza C, Plaza Z, Soriano V (2014). Changes in liver fibrosis in HIV/HCV-coinfected patients following different outcomes with peginterferon plus ribavirin therapy. J Viral Hepat.

[13] Tada T, Kumada T, Toyoda H, Sone Y, Takeshima K, Ogawa S, Goto T, Wakahata A, Nakashima M, Nakamuta M (2018). Viral eradication reduces both liver stiffness and steatosis in patients with chronic hepatitis C virus infection who received direct-acting anti-viral therapy. Aliment Pharmacol Ther.

[14] Lange CM, Bojunga J, Ramos-Lopez E, von Wagner M, Hassler A, Vermehren J, Herrmann E, Badenhoop K, Zeuzem S, Sarrazin C (2011). Vitamin D deficiency and a CYP27B1-1260 promoter polymorphism are associated with chronic hepatitis C and poor response to interferon-alfa based therapy. J Hepatol.

[15] Holick MF, Binkley NC, Bischoff-Ferrari HA, Gordon CM, Hanley DA, Heaney RP, Murad MH, Weaver CM (2011). Evaluation, treatment, and prevention of vitamin D deficiency: an Endocrine Society clinical practice guideline. J Clin Endocrinol Metab.

[16] Castera L, Vergniol J, Foucher J, Le Bail B, Chanteloup E, Haaser M, Darriet M, Couzigou P, De Ledinghen V (2005). Prospective comparison of transient elastography, Fibrotest, APRI, and liver biopsy for the assessment of fibrosis in chronic hepatitis C. Gastroenterology.

[17] Afdhal NH, Bacon BR, Patel K, Lawitz EJ, Gordon SC, Nelson DR, Challies TL, Nasser I, Garg J, Wei LJ (2015). Accuracy of fibroscan, compared with histology, in analysis of liver fibrosis in patients with hepatitis B or C: a United States multicenter study. Clin Gastroenterol Hepatol.

[18] European Association for the Study of the Liver (2018). EASL recommendations on treatment of hepatitis C. J Hepatol.

[19] Cheng JB, Levine MA, Bell NH, Mangelsdorf DJ, Russell DW (2004). Genetic evidence that the human CYP2R1 enzyme is a key vitamin D 25-hydroxylase. Proc Natl Acad Sci U S A.

[20] Zhu JG, Ochalek JT, Kaufmann M, Jones G, Deluca HF (2013). CYP2R1 is a major, but not exclusive, contributor to 25-hydroxyvitamin D production in vivo. Proc Natl Acad Sci U S A.

[21] Everson GT, Shiffman ML, Hoefs JC, Morgan TR, Sterling RK, Wagner DA, Lauriski S, Curto TM, Stoddard A, Wright EC (2012). Quantitative liver function tests improve the prediction of clinical outcomes in chronic hepatitis C: results from the hepatitis C antiviral long-term treatment against cirrhosis trial. Hepatology (Baltimore, MD).

[22] Chailurkit LO, Aekplakorn W, Ongphiphadhanakul B (2011). Regional variation and determinants of vitamin D status in sunshine-abundant Thailand. BMC Public Health.

[23] Wang TJ, Zhang F, Richards JB, Kestenbaum B, van Meurs JB, Berry D, Kiel DP, Streeten EA, Ohlsson C, Koller DL (2010). Common genetic determinants of vitamin D insufficiency: a genome-wide association study. Lancet (London, England).

[24] Thanapirom K, Suksawatamnuay S, Sukeepaisarnjareon W, Tanwandee T, Charatcharoenwitthaya P, Thongsawat S, Leerapun A, Piratvisuth T, Boonsirichan R, Bunchorntavakul C (2017). Genetic variation in the vitamin D pathway CYP2R1 gene predicts sustained HBeAg seroconversion in chronic hepatitis B patients treated with pegylated interferon: a multicenter study. PLoS ONE.

[25] Thanapirom K, Suksawatamnuay S, Sukeepaisarnjaroen W, Tangkijvanich P, Treeprasertsuk S, Thaimai P, Wasitthankasem R, Poovorawan Y, Komolmit P (2017). Vitamin D-related gene polymorphism predict treatment response to pegylated interferon-based therapy in Thai chronic hepatitis C patients. BMC Gastroenterol.

[26] Farnik H, Bojunga J, Berger A, Allwinn R, Waidmann O, Kronenberger B, Keppler OT, Zeuzem S, Sarrazin C, Lange CM (2013). Low vitamin D serum concentration is associated with high levels of hepatitis B virus replication in chronically infected patients. Hepatology (Baltimore, MD).

[27] Hu YC, Wang WW, Jiang WY, Li CQ, Guo JC, Xun YH (2019). Low vitamin D levels are associated with high viral loads in patients with chronic hepatitis B: a systematic review and meta-analysis. BMC Gastroenterol.

[28] Guo GY, Shi YQ, Wang L, Ren X, Han ZY, Guo CC, Cui LN, Wang JB, Zhu J, Wang N (2015). Serum vitamin D level is associated with disease severity and response to ursodeoxycholic acid in primary biliary cirrhosis. Aliment Pharmacol Ther.

[29] Efe C, Kav T, Aydin C, Cengiz M, Imga NN, Purnak T, Smyk DS, Torgutalp M, Turhan T, Ozenirler S (2014). Low serum vitamin D levels are associated with severe histological features and poor response to therapy in patients with autoimmune hepatitis. Dig Dis Sci.

[30] Komolmit P, Charoensuk K, Thanapirom K, Suksawatamnuay S, Thaimai P, Chirathaworn C, Poovorawan Y (2017). Correction of vitamin D deficiency facilitated suppression of IP-10 and DPP IV levels in patients with chronic hepatitis C: a randomised double-blinded, placebo-control trial. PLoS ONE.

[31] Abramovitch S, Dahan-Bachar L, Sharvit E, Weisman Y, Ben Tov A, Brazowski E, Reif S (2011). Vitamin D inhibits proliferation and profibrotic marker expression in hepatic stellate cells and decreases thioacetamide-induced liver fibrosis in rats. Gut.

[32] Komolmit P, Kimtrakool S, Suksawatamnuay S, Thanapirom K, Chattrasophon K, Thaimai P, Chirathaworn C, Poovorawan Y (2017). Vitamin D supplementation improves serum markers associated with hepatic fibrogenesis in chronic hepatitis C patients: a randomized, double-blind, placebo-controlled study. Sci Rep.

[33] Pan JJ, Bao F, Du E, Skillin C, Frenette CT, Waalen J, Alaparthi L, Goodman ZD, Pockros PJ (2018). Morphometry confirms fibrosis regression from sustained virologic response to direct-acting antivirals for hepatitis C. Hepatol Commun.

[34] Dolmazashvili E, Abutidze A, Chkhartishvili N, Karchava M, Sharvadze L, Tsertsvadze T (2017). Regression of liver fibrosis over a 24-week period after completing direct-acting antiviral therapy in patients with chronic hepatitis C receiving care within the national hepatitis C elimination program in Georgia: results of hepatology clinic HEPA experience. Eur J Gastroenterol Hepatol.

[35] Sagir A, Erhardt A, Schmitt M, Haussinger D (2008). Transient elastography is unreliable for detection of cirrhosis in patients with acute liver damage. Hepatology (Baltimore, MD).

[36] Hezode C, Castera L, Roudot-Thoraval F, Bouvier-Alias M, Rosa I, Roulot D, Leroy V, Mallat A, Pawlotsky JM (2011). Liver stiffness diminishes with antiviral response in chronic hepatitis C. Aliment Pharmacol Ther.

[37] Nielsen MJ, Veidal SS, Karsdal MA, Orsnes-Leeming DJ, Vainer B, Gardner SD, Hamatake R, Goodman ZD, Schuppan D, Patel K (2015). Plasma Pro-C3 (N-terminal type III collagen propeptide) predicts fibrosis progression in patients with chronic hepatitis C. Liver Int Off J Int Assoc Study Liver.

