# Supplementary material for: Changes in hepatic fibrosis and vitamin D levels after viral hepatitis C eradication using direct-acting antiviral therapy
Source: BMC Gastroenterol. 2020 Oct 17;20:346. doi: 10.1186/s12876-020-01485-8 (PMC7568415; doi:10.1186/s12876-020-01485-8)
Supplement: Supplementary file 1 — Additional file 1: Supplementary data 1: Study protocol (Original version). [file 12876_2020_1485_MOESM1_ESM.pdf]

SUBMISSION PROPOSAL FOR
INSTITUTIONAL REVIEW BOARD
FACULTY OF MEDICINE, CHULALONGKORN UNIVERSITY
BANGKOK, THAILAND

สารบัญ **หน้า**

แผ่นหน้า	2
1. Background	3
2. Hypothesis and research questions	6
3. Research design and methodology	7
4. References	11
5. Appendix	
5.1 Case record form	12
5.2 Patient information sheet	16
5.3 Informed consent sheet	22

SUBMISSION PROPOSAL FOR

INSTITUTIONAL REVIEW BOARD

FACULTY OF MEDICINE, CHULALONGKORN UNIVERSITY

BANGKOK, THAILAND

ชื่อโครงการ (Proposal Title)

ภาษาไทย การเปรียบเทียบประสิทธิภาพของเครื่องวัดความยืดหยุ่นของตับ อัลตร้าซาวนด์ เครื่องตรวจคลื่นแม่เหล็กไฟฟ้าในการประเมินภาวะพังผืดตับในผู้ป่วยไวรัสตับอักเสบซีและตับคั่งไขมันที่ไม่ได้เกิดจากแอลกอฮอล์ และการเปลี่ยนแปลงของพังผืดตับติดตามไป 1 ปี

ภาษาอังกฤษ Comparison of transient elastography, acoustic radiation force impulse and magnetic resonance imaging for non-invasive staging of liver fibrosis in patients with chronic hepatitis C and nonalcoholic fatty liver disease: 1-year follow-up of fibrosis progression

ชื่อคณะผู้วิจัย (Investigators)

ผู้วิจัยหลัก ผศ.ดร.นพ.ปิยะวัฒน์ โกมลมิศร์

หน่วยโรคระบบทางเดินอาหารและตับ ภาควิชาอายุรศาสตร์ โรงพยาบาลจุฬาลงกรณ์

1. ความสำคัญและที่มาของปัญหา (Background)

การติดเชื้อไวรัสตับอักเสบซีและภาวะตับคั่งไขมันเป็นสาเหตุสำคัญอันดับต้นๆ ของการเกิดโรคตับอักเสบเรื้อรัง ตับแข็งและมะเร็งตับ กระบวนการอักเสบเรื้อรังที่เกิดขึ้นในตับไม่ว่าจากสาเหตุใดก็ตาม จะกระตุ้นให้เกิดการสร้างพังผืดขึ้น หากผู้ป่วยไม่ได้ไปตรวจประเมินและรักษาสาเหตุให้หมดไปตั้งแต่ระยะเริ่มแรก จะทำให้พังผืดที่เกิดขึ้นนี้สะสมมากขึ้นเรื่อยๆ และทำให้เกิดภาวะตับแข็งในที่สุด แต่เดิมการประเมินระยะของพังผืดในตับนี้ต้องอาศัยการเจาะชิ้นเนื้อจากตับ (liver biopsy) มาตรวจทางพยาธิวิทยาด้วยกล้องจุลทรรศน์และถือเป็นวิธี gold standard ที่ยึดถือปฏิบัติกันมานานและยอมรับกันทั่วโลก แต่อย่างไรก็ตามการเจาะชิ้นเนื้อตับอาจจะมีภาวะแทรกซ้อนที่อันตรายตามมาได้ เช่น เลือดออกพบประมาณ 1:2,500 ถึง 1:10,000 และพบอัตราเสียชีวิตประมาณร้อยละ 0.09 อย่างไรก็ตามด้วยการพัฒนาองค์ความรู้ทางการแพทย์ ทำให้ได้มีการคิดค้นเครื่องมือประเมินพังผืดตับที่ไม่ก่อให้เกิดอันตรายแก่ผู้ป่วยขึ้นมาเมื่อประมาณ 7-10 ปีที่ผ่านมา และทำให้เกิดการเปลี่ยนแปลงครั้งใหญ่ของศาสตร์ด้านการแพทย์โรคตับให้เข้าสู่ยุคใหม่ ก่อให้เกิดประโยชน์แก่ผู้ป่วยที่สามารถตรวจพังผืดและตับแข็งได้ตั้งแต่ระยะเริ่มแรก

ในช่วงหลายปีที่ผ่านมาได้มีการพัฒนาเครื่องมือประเมินพังผืดตับนี้อย่างต่อเนื่อง โดยมีการประยุกต์ปรับเปลี่ยนเครื่องตรวจภาพทางรังสีที่มีอยู่เดิม เช่น อัลตร้าซาวนด์ เครื่องเอ็มอาร์ไอหรือคลื่นแม่เหล็กไฟฟ้าให้สามารถประเมินพังผืดในตับได้เช่นกัน การศึกษาเพื่อเปรียบเทียบประสิทธิภาพของเครื่องมือต่างๆเหล่านี้ในการประเมินภาวะพังผืดตับในผู้ป่วยไวรัสตับอักเสบซีและตับคั่งไขมันเรื้อรังยังไม่เคยมีข้อมูลมาก่อนในประเทศไทย จึงเป็นที่มาของงานวิจัยชิ้นนี้

การติดเชื้อไวรัสตับอักเสบซีเป็นปัญหาสาธารณสุขที่ทั่วโลกให้ความสำคัญ เนื่องจากมีประชากรกว่า 185 ล้านคนที่มีการติดเชื้อชนิดนี้(1) ส่วนภาวะตับคั่งไขมันที่ไม่ได้เกิดจากแอลกอฮอล์ (non-alcoholic fatty liver disease) ก็เป็นอีกสาเหตุหนึ่งที่ทำให้เกิดโรคตับอักเสบเรื้อรังโดยเฉพาะประเทศทางชาติตะวันตกโดยเฉลี่ยพบอุบัติการณ์ประมาณร้อยละ 19-46(2) ทั้งไวรัสตับอักเสบซีและภาวะตับคั่งไขมันเป็นสาเหตุที่สำคัญของการเกิดตับอักเสบเรื้อรัง ตับแข็งและมะเร็งตับในประเทศไทย

<u>การเกิดพังผืดในตับและการวินิจฉัย</u>

การเกิดพังผืดในตับ (Hepatic fibrosis) เริ่มต้นจากเกิดการอักเสบจากสาเหตุต่างๆ อาทิเช่น การติดเชื้อ แอลกอฮอล์ สารพิษ ยา แล้วไปกระตุ้น hepatic stellate cells (HSCs) ในภาวะปกติให้กลายเป็น activated HSCs หรือ Myofibroblasts ซึ่งจะทำให้เกิดการหลั่งไซโตไคน์ที่เกี่ยวข้องกับการสร้างพังพืดที่เพิ่มขึ้น (fibrogenic cytokines) เช่น transforming growth factor (TGF-β) , platelet derived growth factor (PDGF) , connective tissue growth factor (CTGF) เกิดการสะสมของ extracellular matrix และการสร้างพังผืด (fibrogenesis) ขึ้นในตับและเมื่อสะสมมากขึ้นจะเกิดตับแข็งในท้ายที่สุด(3, 4) ในปัจจุบันนี้เชื่อว่าพังผืดที่เกิดขึ้นนี้สามารถจะสลายไปได้ (fibrosis regression) หากรักษาการอักเสบที่เป็นต้นเหตุให้หมดไป

การประเมินระยะของพังผืดตับในผู้ป่วยโรคตับจากเหตุต่างๆ มีประโยชน์ในการบอกพยากรณ์โรคในอนาคต เช่นภาวะแทรกซ้อนที่จะเกิดจาก portal hypertension หรือความทุพพลภาพ การเสียชีวิตที่เกิดจากโรคตับ การเจาะชิ้นเนื้อตับเพื่อตรวจทางพยาธิวิทยาเป็นวิธีมาตรฐานที่สากลยอมรับให้ใช้เป็นเครื่องมือในการประเมินระยะของพังผืดในตับ ซึ่งมีผู้เชี่ยวชาญหลายท่านได้เสนอระบบที่ใช้ในการจัดแบ่งระยะของพังผืดตับไว้หลายแบบ ได้แก่ METAVIR, Knodell, Scheuer, Ishak score(5) (แสดงในตาราง 1) ซึ่งมีข้อมูลจากงานศึกษาวิจัยรองรับว่าสอดคล้องไปกับอาการทางคลินิกและการพยากรณ์โรคทั้งในผู้ป่วยตับเรื้อรังจากไวรัสตับอักเสบบี ซี ไขมันคั่งตับและโรคตับอื่นๆ

ตารางที่1 ระบบการประเมินระยะของพังผืดตับในทางพยาธิวิทยา

**METAVIR score**	
**Stage**	
**0**	No fibrosis
**1**	Mild fibrosis-portal fibrosis
**2**	Moderate fibrosis-portal fibrosis and few septa
**3**	Severe fibrosis-numerous septa without cirrhosis
**4**	Cirrhosis
**ISHAK score**	
**Stage**	
**0**	No fibrosis
**1**	Fibrous expansion of **some** portal area ± short fibrous septa
**2**	Fibrous expansion of **most** portal area ± short fibrous septa
**3**	Fibrous expansion of some portal area with occasional portal to portal bridging
**4**	Fibrous expansion of some portal area with marked bridging as well as portal to central
**5**	Marked bridging (portal to portal and/or portal to central) with occasional nodules
**6**	Cirrhosis, probable or definite

อย่างไรก็ตามการเจาะชิ้นเนื้อตับอาจทำให้เกิดภาวะแทรกซ้อนได้ เช่น อาการปวดพบได้มากถึงร้อยละ 84, ภาวะเลือดออกพบเฉลี่ยประมาณ 1:2,500 ถึง 1:10,000 โดยเฉพาะกลุ่มผู้ป่วยที่เสี่ยง เช่น มีภาวะไตวายเรื้อรังร่วมด้วย ตับแข็ง มี coagulopathy, การเสียชีวิตพบเฉลี่ยน้อยกว่า 1: 10,000 ซึ่งมักจะเกิดจากการเสียเลือดอย่างมากโดยเฉพาะในการเจาะชิ้นเนื้อจากรอยโรคที่เป็นก้อนเนื้อในตับ นอกจากนี้ยังมีรายงานภาวะแทรกซ้อนอื่นๆ ที่พบได้ เช่น pneumo/hemothorax, viscous organs perforation, bile peritonitis, การติดเชื้อ (bacteremia, sepsis), hemobilia, neuralgia และ ventricular arrthymia(6) อย่างไรก็ตามการประเมินพยาธิวิทยาจากชิ้นเนื้อตับก็มีข้อจำกัดหลายประการ ยกตัวอย่างเช่น ชิ้นเนื้อที่เจาะมาเพียงส่วนเล็กๆจากตับอาจจะไม่ได้เป็นตัวแทนของเนื้อตับทั้งอัน การสะสมของพังผืดในตับแต่ละตำแหน่งอาจจะไม่เท่ากัน กระบวนการเจาะชิ้นเนื้อเองก็อาจจะเกิด sampling error ขึ้นได้ ขนาดของเข็มที่ใช้เจาะ ความยาวของชิ้นเนื้อที่ได้ออกมาส่งผลในการแปลผล พยาธิแพทย์ที่อ่านผลต่างกันก็จะมี interobserver variation(7) ด้วยเหตุผลหลายประการเหล่านี้จึงเป็นที่มาของการพัฒนาเครื่องมือในการประเมินพังผืดตับที่ไม่ก่อให้เกิดอันตรายแก่ผู้ป่วยในปัจจุบัน

<u>การประเมินพังผืดตับโดยเครื่องมือที่ไม่ก่อให้เกิดอันตรายแก่ผู้ป่วย (non-invasive tests to assess staging of liver fibrosis)</u>

ในปัจจุบันเครื่องมือที่ใช้ประเมินพังผืดในตับโดยไม่ก่อให้เกิดอันตรายแก่ผู้ป่วย จำแนกเป็น 2 ชนิดได้แก่

1. **Serum biomarkers** อาศัยหลักการตรวจ biomarkers ต่างๆ ในเลือดที่เป็นองค์ประกอบของ fibrotic pathways เช่น Fibrotest® (Biopredictive, Paris, France), AST to Platelet Ratio (APRI), Fibrospect® (Promotheus Laboratory Inc, San Diego, USA), Enhanced Liver Fibrosis score® (ELF) (Siemens Healthcare, Erlangen, Germany) เป็นต้น ซึ่งมีข้อมูลงานวิจัยรองรับทั้งจากผู้ป่วยไวรัสตับอักเสบบี ซี ไขมันคั่งตับ(8)

2. Liver stiffness measurement (Liver elastography) อาศัยหลักการวัดความยืดหยุ่นของตับ ซึ่งแบ่งเป็น 2 ชนิดได้แก่

1.) *Static หรือ quasistatic (strain imaging)* เช่น eMode (Hitachi), eSie Touch (Siemens Healthcare), SonixTOUCH (Ultrasonix), Strain-based elastography (Philips Healthcare), Strain elastography (GE healthcare)

2.) *Dynamic (Shear-wave imaging)*

2.1 Transient (Ultrasound-based elastography)

2.1.1 One-dimensional Transient elastrography: Fibroscan (Echosens)

2.1.2 Point shear wave elastography (pSWE) หรือ acoustic radiation force impulse imaging (ARFI) ได้แก่ Virtual touch tissue quantification™, (Siemens Healthcare), elastography point quantification (ElastPQ™, Philips), shear wave elastography (GE healthcare)

2.1.3 Two-dimensional shear wave elastography (2D-SWE) ได้แก่ Aixplorer™ (Supersonic Imagine, France)

2.2 Continuous ได้แก่ Magnetic resonance elastography (MRE)(GE healthcare, Philips Healthcare, Siemens Healthcare)

2. วัตถุประสงค์ของการวิจัย (Objectives)

Primary endpoint

เพื่อเปรียบเทียบการวัดระยะพังผืดในตับในผู้ป่วยไวรัสตับอักเสบซีเรื้อรังและตับคั่งไขมันโดยใช้เครื่องมือ Fibroscan, acoustic radiation force impulse imaging และ magnetic resonance elastography

Secondary endpoint

1. เพื่อประเมินระยะพังผืดในตับในผู้ป่วยไวรัสตับอักเสบซีเรื้อรังและตับคั่งไขมันที่เปลี่ยนแปลงไปในระยะเวลา 1 ปีโดยใช้เครื่องมือ Fibroscan, acoustic radiation force impulse imaging และ magnetic resonance elastography
2. เพื่อประเมินคุณภาพชีวิตของผู้ป่วยตับอักเสบเรื้อรังจากไวรัสซีและตับคั่งไขมัน

3. คำถามของการวิจัย/สมมติฐาน (Hypothesis)

คำถามหลัก

การตรวจวัดระยะพังผืดในตับในผู้ป่วยไวรัสตับอักเสบซีเรื้อรังและตับคั่งไขมันโดยใช้เครื่องมือ Fibroscan, acoustic radiation force impulse imaging และ magnetic resonance elastography มีความแตกต่างกันอย่างไร

คำถามรอง

1. เมื่อติดตามไป 1 ปีระยะพังผืดในตับในผู้ป่วยไวรัสตับอักเสบซีเรื้อรังและตับคั่งไขมันมีความเปลี่ยนแปลงไปอย่างไรเมื่อวัดโดยใช้เครื่องมือ Fibroscan, acoustic radiation force impulse imaging และ magnetic resonance elastography
2. คุณภาพชีวิตของผู้ป่วยตับอักเสบเรื้อรังจากไวรัสซีและตับคั่งไขมันเป็นอย่างไร

4. คำสำคัญ (Keywords)

Chronic hepatitis C infection, non-alcoholic fatty liver diease, Elastography

5. รูปแบบการวิจัย (Research Design)

Prospective, analytic study

6. ระเบียบวิธีการวิจัย (Research Methodology)

ประชากรเป้าหมาย (Target population)

คือ ผู้ป่วยที่มีโรคไวรัสตับอักเสบซี และ ไขมันคั่งตับ

เกณฑ์การคัดเลือกอาสาสมัครเข้าร่วมโครงการวิจัย (Inclusion criteria)

1. ผู้ป่วยได้รับการวินิจฉัยว่าติดเชื้อไวรัสตับอักเสบซีเรื้อรัง โดยมี anti-HCV เป็นบวกและตรวจพบ HCV RNA มากกว่า 6 เดือน
2. อายุตั้งแต่ 18 ปีขึ้นไป

เกณฑ์การไม่รับเข้าโครงการวิจัย (Exclusion Criterias)

1. มีประวัติทานเหล้า > 14 หน่วยต่อสัปดาห์ในเพศชาย หรือ > 7 หน่วยต่อสัปดาห์ในเพศหญิง
2. ผู้ป่วยที่มีโรคติดเชื้อ HIV ร่วมด้วย
3. ผู้ป่วยที่มีโรคติดเชื้อไวรัสตับอักเสบบีร่วมด้วยโดยวินิจฉัยจาก ผล HBsAg เป็นผลบวก
4. ผู้ป่วยที่ไม่ต้องการเข้าร่วมการศึกษาวิจัย
5. ผู้ป่วยที่มีโรคตับอักเสบเรื้อรังจากเหตุอื่นๆ เช่น autoimmune hepatitis, hemochromatosis, Wilson's disease, cholestatic หรือ vascular liver diseases
6. ผู้ป่วย decompensated cirrhosis
7. มีข้อห้ามของการทำ magnetic resonance imaging เช่น ภาวะกลัวที่แคบ (claustrophobia), มีฝังโลหะในร่างกาย, ตั้งครรภ์

<u>วิธีการวิจัย/วิธีดำเนินการวิจัย</u>

1. ชี้แจงวัตถุประสงค์ ขั้นตอนการวิจัย ประโยชน์ที่ผู้ป่วยจะได้รับ รวมถึงผลข้างเคียงที่อาจเกิดขึ้น โดยการอธิบายรายละเอียดกับผู้ป่วยทุกขั้นตอนตั้งแต่วัตถุประสงค์ ขั้นตอนการวิจัย ประโยชน์ที่ผู้ป่วยจะได้รับ รวมถึงผลข้างเคียงที่อาจเกิดขึ้นได้แก่ผู้ป่วยทุกรายด้วยตัวผู้วิจัยเองทุกคน และได้ขอความยินยอมจากผู้ป่วยเป็นลายลักษณ์อักษรทุกครั้งก่อนเริ่มทำงานวิจัย
2. ซักประวัติ ตรวจร่างกายตามแบบบันทึกข้อมูล และเก็บข้อมูลตามแบบสอบถาม

3. ขอเจาะเลือดผู้ป่วยจำนวน 6 ซีซี ใส่ clotted tube และ EDTA เพื่อส่งตรวจการทำงานของตับ (Liver function tests) ตรวจนับเม็ดเลือด (CBC: complete blood count)

4. ตั้งตัวอย่างเลือดไว้ 30 นาทีที่อุณหภูมิห้อง

5.นำตัวอย่างเลือดมาปั่น 15 นาที

6. เก็บแยกซีรั่มไว้ที่อุณหภูมิ $\leq$ - 70 °c

7. ตรวจพังผืดในตับด้วยเครื่องมือ 3 ชนิดคือ Fibroscan, acoustic radiation force impulse imaging และ magnetic resonance elastography

การจัดการกับตัวอย่างทางชีวภาพที่เหลือ

หลังจากเสร็จสิ้นงานวิจัย จะมีการเก็บตัวอย่างเลือดและ DNA ไว้ที่หน่วยโรคระบบทางเดินอาหาร เลขที่ 1873 ตึกภูมิสิริฯ ชั้น 2 สาขาวิชาโรคทางเดินอาหาร โรงพยาบาลจุฬาลงกรณ์ ถนนพระรามสี่ ปทุมวัน กรุงเทพ 10330 เพื่อใช้ในการตรวจคสวามหลากหลายทางชีวภาพตัวอื่นในอนาคต โดยจะเก็บไว้นาน 15 ปี จึงจะทำลาย ถ้ามีการนำตัวอย่างเหล่านี้มาใช้จะมีการเขียนเพื่อขอคำอนุญาตจากคณะกรรมการพิจารณาจริยธรรมการวิจัยก่อน

ขนาดตัวอย่าง และ การคำนวณ

จำนวนตัวอย่างในแต่ละกลุ่ม $= \frac{[Z_{\alpha/2} + Z_{\beta}]^2 \sigma^2}{(d)^2}$

โดย $\alpha = 0.05 \rightarrow Z_{\alpha/2} = 1.96$

$\beta = 0.1 \rightarrow Z_{\beta} = 1.28$

$\sigma^2 =$ variance of difference $= SD1^2 + SD2^2 - 2(r)SD1SD2$

d = mean of difference = (X1 – X2)

ในผู้ป่วยไวรัสตับอักเสบซีจากการศึกษาเก่าพบว่า

X1 = ค่า mean ของพังผืดตับในระยะ advanced fibrosis เมื่อตรวจด้วยเครื่อง TE = 7.1

X2 = ค่า mean ของบงพังผืดตับในระยะ advanced fibrosis เมื่อตรวจด้วยเครื่อง MRE = 4.15

SD1 = SD ของพังผืดตับเมื่อตรวจด้วยเครื่อง TE = 0.54

SD1 = SD ของพังผืดตับเมื่อตรวจด้วยเครื่อง TE = 8.2

r = correlation ระหว่าง TE และ MRE = 0

$\sigma^2 = (0.54)^2 + (8.2)^2 - 2(0)(0.54)(8.2) = 67.5$

เมื่อแทนค่าลงไปในสูตร

จำนวนตัวอย่างในแต่ละกลุ่ม $= \frac{[1.96 + 1.28]^2 \, 67.5}{(7.1 - 4.15)^2} = 81.4$

และเมื่อติดตามไป 1 ปีถ้าประมาณมีผู้ป่วยที่ loss to follow- up ประมาณร้อยละ 20 เพราะฉะนั้นต้องมีผู้ป่วยไวรรัสตับอักเสบซีประมาณ 98 ราย

7. การรวบรวมข้อมูล (Data Collection)

เก็บข้อมูลโดยแพทย์และเจ้าหน้าที่วิจัย หน่วยทางเดินอาหารและตับ โรงพยาบาลจุฬาลงกรณ์

8. การวิเคราะห์ข้อมูลและสถิติที่ใช้วิเคราะห์ (Data Analysis and Statistics)

การวิเคราะห์ข้อมูลในส่วนของข้อมูลเชิงคุณภาพ (Qualitative or categorical data) เป็นจำนวน (Frequency), ร้อยละ (Percentage) และข้อมูลเชิงปริมาณ (Quantitative data) นำเสนอเป็น ค่าเฉลี่ย (mean) ค่ามัธยฐาน (median) ค่าเบี่ยงเบนมาตรฐาน (Standard deviation: SD)

Repeated ANOVA ใช้เพื่อวิเคราะห์หาความแตกต่างของค่าเฉลี่ยพังผืดที่แตกต่างกันจากเครื่องมือทั้ง 3 ชนิด ค่า P value ที่น้อยกว่า 0.05 ถือเป็นค่าที่มีนัยสำคัญทางสถิติ

งานวิจัยนี้วิเคราะห์ข้อมูลโดยใช้ MRE เป็น gold standard เนื่องจากข้อมูลการศึกษาที่ยอมรับการใช้เครื่องมือชนิดนี้เป็น gold standard แทนการเจาะชิ้นเนื้อตับ

9. ประโยชน์ที่คาดว่าจะได้รับจากการวิจัย (Expected or Anticipated Benefit Gain)

เนื่องจากการศึกษาในเรื่องนี้ยังไม่เคยมีมาก่อนในประเทศไทยและเครื่องมือการตรวจวัดพังผืดถือเป็นเครื่องมือใหม่ที่นานาชาติให้ความสนใจที่จะมาทดแทนการเจาะชิ้นเนื้อตับ และได้ประโยชน์ใช้ประยุกต์ไปสู่การดูแลรักษาผู้ป่วยโรคตับอักเสบเรื้อรังซึ่งมีอยู่เป็นจำนวนมากเนื่องจากเป็นเครื่องมือตรวจวัดที่ไม่ก่อให้เกิดอันตรายแก่ผู้ป่วย อีกทั้งผู้ป่วยจะได้ประโยชน์จากงานวิจัยในการทราบข้อมูลระดับพังผืดตับเมื่อติดตามไป 1 ปีว่ามีการเปลี่ยนแปลงไปมากเพียงใด

10. อุปสรรคที่อาจจะเกิดขึ้น (Challenges)

ปัญหาในการเรื่องการเก็บดูแล specimen และการเจาะเลือด ได้แก้ไขโดยการจ้างเจ้าหน้าที่ห้องปฏิบัติการและพยาบาลผู้เชี่ยวชาญให้ดูแลโดยตรง

11. ความเสี่ยงที่จะเกิดขึ้นและความรับผิดชอบ (Risk and Investigator’s Responsibility)

ผู้เข้าร่วมวิจัยอาจได้รับความเสี่ยง เช่น เสียเวลา ไม่สะดวก ไม่สบายจากการเจาะเลือดเพิ่มเติม แพทย์ผู้ทำการวิจัยขอชี้แจงถึงความเสี่ยงและความไม่สบายที่อาจเกิดได้จากการเข้าร่วมการศึกษานี้ และหากท่านมีข้อสงสัยใดๆ เกี่ยวกับความเสี่ยงที่อาจได้รับจากการเข้าร่วมในโครงการวิจัย ท่านสามารถสอบถามจากผู้ทำวิจัยได้ตลอดเวลา

12. ระยะเวลาที่ใช้ในการวิจัยตั้งแต่เริ่มต้นจนสิ้นสุดโครงการ (Timeline)

ระยะเวลาทำการศึกษา 12 เดือน

13. สถานที่ทำวิจัย (Venue of the Study)

คลินิกโรคตับ หน่วยทางเดินอาหาร โรงพยาบาลจุฬาลงกรณ์

14. การบริหารงานวิจัยและตารางการปฏิบัติงาน (Tabulation of Research Activities and Timeline)

รายการ	2559							2560			
	06	07	08	09	10	11	12	01	02	03	04
รวบรวมข้อมูล และขอจริยธรรม	■	■	■	■	■	■					
รวบรวมsampleจากผู้เข้าร่วมโครงการ							■	■			
Assessment serum AFP, AFP-L3, PIVKAII									■		
รวบรวมข้อมูล									■	■	
วิเคราะห์ข้อมูล									■	■	
สรุปผลการศึกษาและรายงาน									■	■	
Manuscript and send for publications											■

15. งบประมาณ (Budget)

**รายการ**	**รายละเอียดของอัตราค่าใช้จ่าย x รายการ**	**รวม**
1. หมวดค่าจ้างชั่วคราว		
1.1 นักวิทยาศาสตร์ (ปริญญาโท)	จำนวน 1 คน 15,000 ต่อเดือน x 2 เดือน	30,000
	**รวมรายการ 1. หมวดค่าจ้างชั่วคราว**	**30,000**
2. หมวดค่าใช้สอย		
2.1 ค่าใช้จ่ายกระบวนการเก็บตัวอย่างและข้อมูล	300 บาท x 196 คน	58,800
	**รวมรายการ 2. หมวดค่าใช้สอย**	**58,800**
3. ค่าตอบแทน		
3.1 ค่าตอบแทนผู้ช่วยวิจัย	จำนวน 1 คน x 2000 บาท x 12 เดือน	24,000
	**รวมรายการ 3. หมวดค่าตอบแทน**	**24,000**
4. ค่าวัสดุและสาธารณูปโภค		

4.1 วัสดุสำนักงาน		5,000
4.2 วัสดุคอมพิวเตอร์		10,000
4.4 ค่าวัสดุทางวิทยาศาสตร์		
4.4.1 ค่าจัดแยก buffy coat and serum	196 ราย x 200 บาท	39,200
4.4.2 ค่าตรวจแยก DNA	196 ราย x 200 บาท	39,200
4.4.3 ตรวจ SNPs ด้วย RFLP และ Genotyping	196 ราย x 400 บาท	78,400
	**รวมรายการ 4. หมวดวัสดุและสาธารณูประโภค**	**156,800**
**TOTAL**		**268,800**

16. References

1. Mohd Hanafiah K, Groeger J, Flaxman AD, Wiersma ST. Global epidemiology of hepatitis C virus infection: new estimates of age-specific antibody to HCV seroprevalence. Hepatology 2013;57:1333-1342.
2. Williams CD, Stengel J, Asike MI, Torres DM, Shaw J, Contreras M, Landt CL, et al. Prevalence of nonalcoholic fatty liver disease and nonalcoholic steatohepatitis among a largely middle-aged population utilizing ultrasound and liver biopsy: a prospective study. Gastroenterology 2011;140:124-131.
3. Lee YA, Wallace MC, Friedman SL. Pathobiology of liver fibrosis: a translational success story. Gut 2015;64:830-841.
4. Urban ML, Manenti L, Vaglio A. Fibrosis--A Common Pathway to Organ Injury and Failure. N Engl J Med 2015;373:95-96.
5. Bedossa P, Poynard T. An algorithm for the grading of activity in chronic hepatitis C. The METAVIR Cooperative Study Group. Hepatology 1996;24:289-293.
6. Rockey DC, Caldwell SH, Goodman ZD, Nelson RC, Smith AD. Liver biopsy. Hepatology 2009;49:1017-1044.
7. Rousselet MC, Michalak S, Dupre F, Croue A, Bedossa P, Saint-Andre JP, Cales P. Sources of variability in histological scoring of chronic viral hepatitis. Hepatology 2005;41:257-264.
8. Fontana RJ, Goodman ZD, Dienstag JL, Bonkovsky HL, Naishadham D, Sterling RK, Su GL, et al. Relationship of serum fibrosis markers with liver fibrosis stage and collagen content in patients with advanced chronic hepatitis C. Hepatology 2008;47:789-798.

1. Mohd Hanafiah K, Groeger J, Flaxman AD, Wiersma ST. Global epidemiology of hepatitis C virus infection: new estimates of age-specific antibody to HCV seroprevalence. Hepatology 2013;57:1333-1342.
2. Williams CD, Stengel J, Asike MI, Torres DM, Shaw J, Contreras M, Landt CL, et al. Prevalence of nonalcoholic fatty liver disease and nonalcoholic steatohepatitis among a largely middle-aged population utilizing ultrasound and liver biopsy: a prospective study. Gastroenterology 2011;140:124-131.
3. Lee YA, Wallace MC, Friedman SL. Pathobiology of liver fibrosis: a translational success story. Gut 2015;64:830-841.
4. Urban ML, Manenti L, Vaglio A. Fibrosis--A Common Pathway to Organ Injury and Failure. N Engl J Med 2015;373:95-96.
5. Bedossa P, Poynard T. An algorithm for the grading of activity in chronic hepatitis C. The METAVIR Cooperative Study Group. Hepatology 1996;24:289-293.
6. Rockey DC, Caldwell SH, Goodman ZD, Nelson RC, Smith AD. Liver biopsy. Hepatology 2009;49:1017-1044.
7. Rousselet MC, Michalak S, Dupre F, Croue A, Bedossa P, Saint-Andre JP, Cales P. Sources of variability in histological scoring of chronic viral hepatitis. Hepatology 2005;41:257-264.
8. Fontana RJ, Goodman ZD, Dienstag JL, Bonkovsky HL, Naishadham D, Sterling RK, Su GL, et al. Relationship of serum fibrosis markers with liver fibrosis stage and collagen content in patients with advanced chronic hepatitis C. Hepatology 2008;47:789-798.
9. Kim SU, Choi GH, Han WK, Kim BK, Park JY, Kim DY, Choi JS, et al. What are 'true normal' liver stiffness values using FibroScan?: a prospective study in healthy living liver and kidney donors in South Korea. Liver Int 2010;30:268-274.
10. Castera L, Vergniol J, Foucher J, Le Bail B, Chanteloup E, Haaser M, Darriet M, et al. Prospective comparison of transient elastography, Fibrotest, APRI, and liver biopsy for the assessment of fibrosis in chronic hepatitis C. Gastroenterology 2005;128:343-350.
11. Zarski JP, Sturm N, Guechot J, Paris A, Zafrani ES, Asselah T, Boisson RC, et al. Comparison of nine blood tests and transient elastography for liver fibrosis in chronic hepatitis C: the ANRS HCEP-23 study. J Hepatol 2012;56:55-62.
12. Wong VW, Vergniol J, Wong GL, Foucher J, Chan HL, Le Bail B, Choi PC, et al. Diagnosis of fibrosis and cirrhosis using liver stiffness measurement in nonalcoholic fatty liver disease. Hepatology 2010;51:454-462.
13. Sporea I, Bota S, Peck-Radosavljevic M, Sirli R, Tanaka H, Iijima H, Badea R, et al. Acoustic Radiation Force Impulse elastography for fibrosis evaluation in patients with chronic hepatitis C: an international multicenter study. Eur J Radiol 2012;81:4112-4118.

14. Liu H, Fu J, Hong R, Liu L, Li F. Acoustic Radiation Force Impulse Elastography for the Non-Invasive Evaluation of Hepatic Fibrosis in Non-Alcoholic Fatty Liver Disease Patients: A Systematic Review & Meta-Analysis. PLoS One 2015;10:e0127782.
15. Fierbinteanu Braticevici C, Sporea I, Panaitescu E, Tribus L. Value of acoustic radiation force impulse imaging elastography for non-invasive evaluation of patients with nonalcoholic fatty liver disease. Ultrasound Med Biol 2013;39:1942-1950.
16. Shi Y, Xia F, Li QJ, Li JH, Yu B, Li Y, An H, et al. Magnetic Resonance Elastography for the Evaluation of Liver Fibrosis in Chronic Hepatitis B and C by Using Both Gradient-Recalled Echo and Spin-Echo Echo Planar Imaging: A Prospective Study. Am J Gastroenterol 2016.
17. Wu WP, Chou CT, Chen RC, Lee CW, Lee KW, Wu HK. Non-Invasive Evaluation of Hepatic Fibrosis: The Diagnostic Performance of Magnetic Resonance Elastography in Patients with Viral Hepatitis B or C. PLoS One 2015;10:e0140068.
18. Kishore R, Loomba R, Sterling RK. Editorial: A New Spin on Magnetic Resonance Elastography. Am J Gastroenterol 2016;111:834-837.
19. Shiha G, Ibrahim A, Helmy A, Sarin SK, Omata M, Kumar A, Bernstien D, et al. Asian-Pacific Association for the Study of the Liver (APASL) consensus guidelines on invasive and non-invasive assessment of hepatic fibrosis: a 2016 update. Hepatol Int 2016.
20. Loomba R, Cui J, Wolfson T, Haufe W, Hooker J, Szeverenyi N, Ang B, et al. Novel 3D Magnetic Resonance Elastography for the Noninvasive Diagnosis of Advanced Fibrosis in NAFLD: A Prospective Study. Am J Gastroenterol 2016;111:986-994.

**โครงการ "รู้ทันตับแข็ง จากไวรัสตับอักเสบ ซี และไขมันตับ"**<br><br>ศูนย์ความเป็นเลิศทางการแพทย์ด้านโรคตับ แห่งโรงพยาบาลจุฬาลงกรณ์ ร่วมกับ หน่วยทางเดินอาหาร ฝ่ายอายุรศาสตร์ และฝ่ายรังสีวิทยา<br><br>Case record form	Patient code : ☐☐☐<br>Date of data record : (Day-Month-Year)<br>☐☐-☐☐-☐☐☐☐

ส่วนที่ 1: ข้อมูลพื้นฐาน

1. เพศ ☐1. ชาย ☐ 2.หญิง 2. วันเกิด _____ / _______ / ___________

3. เบอร์โทรติดต่อ บ้าน: _______- ______________ มือถือ: _______- ______________

4. ที่อยู่____

5. ภูมิลำเนาจังหวัด ____________

6. น้ำหนัก___________ กิโลกรัม

7. ส่วนสูง__________________ เซนติเมตร

8. เส้นรอบเอว__________ เซนติเมตร

9. ประวัติโรคประจำตัว (ตอบได้มากกว่า 1ข้อ)

☐ 1.ไม่ทราบ/ไม่มีข้อมูล ☐ 2.ไม่มีโรคประจำตัว

☐ 3. มีโรคประจำตัว (ระบุ)

☐ 3.1. หลอดเลือดหัวใจ ☐ 3.2 โรคไต ☐ 3.3 ไขมันโลหิตสูง

☐ 3.4 เบาหวาน ☐ 3.5 ความดันโลหิตสูง ☐ 3.6 อื่นๆ (ระบุ)_______________

10. ประวัติการดื่มเหล้า ☐ 1.ดื่ม ☐ 2. เคยดื่ม (หยุด > 6เดือน) ☐ 3. ไม่ดื่ม ☐9.ไม่มีข้อมูล

11. ประวัติการได้รับเลือด ☐ 1. มี ระบุเมื่อปี พ.ศ. ___________ ☐ 2. ไม่มี ☐9.ไม่มีข้อมูล

12. สัก tattoo เพียซซิ่ง ☐ 1. มี ☐ 2. ไม่มี ☐9.ไม่มีข้อมูล

13. ประวัติเคยใช้ยาเสพติดฉีดเข้าเส้น ☐ 1. มี ☐ 2. ไม่มี ☐9.ไม่มีข้อมูล

14. ประวัติไวรัสตับอักเสบ มะเร็งตับในครอบครัว ☐ 1. มี ☐ 2. ไม่มี ☐9.ไม่มีข้อมูล

ส่วนที่ 2: ข้อมูลการวินิจฉัย

15. การวินิจฉัยสาเหตุ ปี พ.ศ.______________

☐ 1.ไวรัสซี genotype ☐ 1. G1 ☐ 2. G 2 ☐ 3. G 3 ☐ 4. G 6 ☐5.อื่นๆระบุ____ ☐9.ไม่มีข้อมูล

การรักษาที่เคยได้รับ ☐ 1. ไม่เคยรักษา ☐ 2. เคยรักษาด้วย______________________

☐ 2.ไขมันเกาะตับ

การรักษาที่เคยได้รับ ☐ 1. ไม่เคยรักษา ☐ 2. เคยรักษาด้วย______________________

16. ระยะโรค ☐ 1. Chronic hepatitis ☐ 2. Cirrhosis ☐ 3. Not assess

17. Fibroscan Date______________ ☐ 1. CAP__________dB/m ☐ 2. TE_____________Kpa

18. มะเร็งตับ ☐ 1 มี ☐ 2. ไม่มี

ส่วนที่ 3: ผลเลือดก่อนเข้าโครงการ Date of collected specimen ☐☐ - ☐☐ - ☐☐☐☐ 19.

CBC: Hb_______ Hct _______ WBC_______ (%neu_____) Plt _______ INR _______ PT_______

20. Liver function tests: TB________ DB ________ AST ________ ALT ________ ALP________ Alb ________

Glb________

21. BUN ________ Cr________

22. FBS_______________ HbA1C_______________ mg%

TG_______________ LDL_______________ CHol_______________ HDL_______________

23. Viral hepatitis

1. Anti-HCV ☐1. Positive ☐ 2. Negative ☐ 9.ไม่มีข้อมูล

2. HCV-viral load ☐ 1. ทำ (ระบุ) ☐☐ , ☐☐☐ , ☐☐☐ (IU/ml)

☐ 2. ไม่ทำ ☐ 9. ไม่มีข้อมูล

3. HCV Genotype ☐1.ทำ ☐ 2.ไม่ทำ ☐ 9.ไม่มีข้อมูล

3.1 ☐ 1. G1 ☐ 2. G 2 ☐3. G 3 ☐4. G 6 ☐5.อื่นๆระบุ____ ☐9.ไม่มีข้อมูล

4. HBsAg ☐1. Positive ☐ 2. Negative ☐ 9.ไม่มีข้อมูล

5. Anti-HIV ☐1. Positive ☐ 2. Negative ☐ 9.ไม่มีข้อมูล

ส่วนที่ 4: ผลการประเมินพังผืดตับ

24. Fibroscan Date_________________ Probe ☐ 1. M ☐ 2. XL Success rate_________% IQR__________%

☐ 1. CAP___________dB/m ☐ 2. TE_______________Kpa

25. USG Date_________________☐ 1. TE_______________Kpa Success ☐ 1. Yes ☐ 2. No

26. MRE Date_________________☐ 1. TE_______________Kpa Success ☐ 1. Yes ☐ 2. No

**** นัดเข้าคลินิกไวรัสซี วันที่ ☐☐ - ☐☐ - ☐☐☐☐**

ส่วนที่ 5: ผลเลือดและการประเมินพังผืดตับติดตามที่ 1 ปี

Date of collected specimen ☐☐ - ☐☐ - ☐☐☐☐

27. CBC: Hb________ Hct ________ WBC________ (%neu______) Plt ________ INR ________ PT________

28. Liver function tests: TB________ DB ________ AST ________ ALT ________ ALP________ Alb ________

Glb________

29. BUN ________ Cr________

30. FBS_______________ HbA1C_______________ mg%

TG_______________ LDL_______________ CHol_______________ HDL_______________

31. Fibroscan Date_________________ Probe ☐ 1. M ☐ 2. XL Success rate_________% IQR__________%

☐ 1. CAP___________dB/m ☐ 2. TE_______________Kpa

32. USG Date_________________☐ 1. TE_______________Kpa Success ☐ 1. Yes ☐ 2. No

33. MRE Date_________________☐ 1. TE_______________Kpa Success ☐ 1. Yes ☐ 2. No

34. ผลการรักษาไวรัสซี ☐ 1. ไม่ได้รักษา

☐ 2. รักษา ด้วยยา____________ เวลา _____เดือน

☐ 1. หาย ☐ 2. ไม่หาย

<u>ข้อมูลสำหรับผู้เข้าร่วมในโครงการวิจัย</u>

<u>ชื่อโครงการวิจัย</u> การเปรียบเทียบประสิทธิภาพของเครื่องวัดความยืดหยุ่นของตับ อัลตร้าซาวนด์ เครื่องตรวจคลื่นแม่เหล็กไฟฟ้าในการประเมินภาวะพังผืดตับในผู้ป่วยไวรัสตับอักเสบซีและตับคั่งไขมันที่ไม่ได้เกิดจากแอลกอฮอล์ และการเปลี่ยนแปลงของพังผืดตับติดตามไป ๑ ปี

<u>แพทย์ผู้ทำวิจัย</u>

ชื่อ ผศ.ดร.นพ.ปิยะวัฒน์ โกมลมิศร์

ที่อยู่ หน่วยโรคระบบทางเดินอาหารและตับ ภาควิชาอายุรศาสตร์ คณะแพทยศาสตร์ โรงพยาบาลจุฬาลงกรณ์

เบอร์โทรศัพท์ โทร. ๐๒-๒๕๖-๔๒๖๕ (ในเวลาราชการ), ๐๙๐-๖๗๖๔๘๒๔ (ตลอด ๒๔ ชั่วโมง)

เรียน ผู้เข้าร่วมโครงการวิจัยทุกท่าน

ท่านได้รับเชิญให้เข้าร่วมในโครงการวิจัยนี้เนื่องจากท่านได้รับการวินิจฉัยว่าเป็นโรคตับอักเสบเรื้อรัง โครงการวิจัยนี้เป็นเพียงการตรวจหามะเร็งตับ

ก่อนที่ท่านจะตัดสินใจเข้าร่วมในการศึกษาวิจัยดังกล่าว ขอให้ท่านอ่านเอกสารฉบับนี้อย่างถี่ถ้วน เพื่อให้ท่านได้ทราบถึงเหตุผลและรายละเอียดของการศึกษาวิจัยในครั้งนี้ หากท่านมีข้อสงสัยใดๆ เพิ่มเติม กรุณาซักถามจากทีมงานของแพทย์ผู้ทำวิจัย หรือแพทย์ผู้ร่วมทำวิจัยซึ่งจะเป็นผู้สามารถตอบคำถามและให้ความกระจ่างแก่ท่านได้

ท่านสามารถขอคำแนะนำในการเข้าร่วมโครงการวิจัยนี้จากครอบครัว เพื่อน หรือแพทย์ประจำตัวของท่านได้ ท่านมีเวลาอย่างเพียงพอในการตัดสินใจโดยอิสระ ถ้าท่านตัดสินใจแล้วว่าจะเข้าร่วมในโครงการวิจัยนี้ ขอให้ท่านลงนามในเอกสารแสดงความยินยอมของโครงการวิจัยนี้

<u>เหตุผลความเป็นมา</u>

การติดเชื้อไวรัสตับอักเสบซีและภาวะตับคั่งไขมันเป็นสาเหตุสำคัญอันดับต้นๆ ของการเกิดโรคตับอักเสบเรื้อรัง ตับแข็งและมะเร็งตับ กระบวนการอักเสบเรื้อรังที่เกิดขึ้นในตับไม่ว่าจากสาเหตุใดก็ตาม จะกระตุ้นให้เกิดการสร้างพังผืดขึ้น หากผู้ป่วยไม่ได้ไปตรวจประเมินและรักษาสาเหตุให้หมดไปตั้งแต่ระยะเริ่มแรก จะทำให้พังผืดที่เกิดขึ้นนี้สะสมมากขึ้นเรื่อยๆ และทำให้เกิดภาวะตับแข็งในที่สุด แต่เดิมการประเมินระยะของพังผืดในตับนี้ต้องอาศัยการเจาะชิ้นเนื้อจากตับ (liver biopsy) มาตรวจทางพยาธิวิทยาด้วยกล้องจุลทรรศน์และถือเป็นวิธีการตรวจที่เป็นมาตรฐานสูงสุด (gold standard) ที่ยึดถือปฏิบัติกันมานานและยอมรับกันทั่วโลก แต่อย่างไรก็ตามการเจาะชิ้นเนื้อตับอาจจะมีภาวะแทรกซ้อนที่อันตรายตามมาได้ เช่น เลือดออกพบประมาณ ๑:๒,๕๐๐ ถึง ๑:๑๐,๐๐๐ และพบอัตราเสียชีวิตประมาณร้อยละ ๐.๐๙ อย่างไรก็ตามด้วยการพัฒนาองค์ความรู้ทางการแพทย์ทำให้ได้มีการคิดค้นเครื่องมือประเมินพังผืดตับที่ไม่ก่อให้เกิดอันตรายแก่ผู้ป่วยขึ้นมาเมื่อประมาณ ๗-๑๐ ปีที่ผ่านมา และทำให้เกิดการเปลี่ยนแปลงครั้งใหญ่ของศาสตร์ด้านการแพทย์โรคตับให้เข้าสู่ยุคใหม่ ก่อให้เกิดประโยชน์แก่ผู้ป่วยที่สามารถตรวจพังผืดและตับแข็งได้ตั้งแต่ระยะเริ่มแรก

ในช่วงหลายปีที่ผ่านมาได้มีการพัฒนาเครื่องมือประเมินพังผืดตับนี้อย่างต่อเนื่อง โดยมีการประยุกต์ปรับเปลี่ยนเครื่องตรวจภาพทางรังสีที่มีอยู่เดิม เช่น อัลตร้าซาวนด์ เครื่องเอ็มอาร์ไอหรือคลื่นแม่เหล็กไฟฟ้าให้สามารถประเมินพังผืดในตับได้เช่นกัน การศึกษาเพื่อเปรียบเทียบประสิทธิภาพของเครื่องมือต่างๆเหล่านี้ในการประเมินภาวะพังผืดตับในผู้ป่วยไวรัสตับอักเสบซีและตับคั่งไขมันเรื้อรังยังไม่เคยมีข้อมูลมาก่อนในประเทศไทย จึงเป็นที่มาของงานวิจัยชิ้นนี้

<u>วัตถุประสงค์ของการศึกษา</u>

วัตถุประสงค์หลัก (Primary endpoint)

เพื่อเปรียบเทียบการวัดระยะพังผืดในตับในผู้เข้าร่วมวิจัยที่เป็นไวรัสตับอักเสบซีเรื้อรังและตับคั่งไขมันโดยใช้เครื่องมือวัดความยืดหยุ่นของตับหรือไฟโบรสแกน อัลตร้าซาวนด์ เครื่องตรวจคลื่นแม่เหล็กไฟฟ้า

วัตถุประสงค์รอง (Secondary endpoint)

๓. เพื่อประเมินระยะพังผืดในตับในผู้เข้าร่วมวิจัยที่เป็นไวรัสตับอักเสบซีเรื้อรังและตับคั่งไขมันที่เปลี่ยนแปลงไปในระยะเวลา ๑ ปีโดยใช้เครื่องมือวัดความยืดหยุ่นของตับหรือไฟโบรสแกน อัลตร้าซาวนด์ เครื่องตรวจคลื่นแม่เหล็กไฟฟ้า

๔. เพื่อประเมินคุณภาพชีวิตของผู้เข้าร่วมวิจัยที่มีตับอักเสบเรื้อรังจากไวรัสซีและตับคั่งไขมัน

แหล่งทุน

ผู้เข้าร่วมงานวิจัยนี้เป็นส่วนหนึ่งของโครงการ “84 พัสสะ พระผู้ให้” เฉลิมพระเกียรติสมเด็จพระนางเจ้าสิริกิติ์ พระบรมราชินีนาถ ของโรงพยาบาลจุฬาลงกรณ์ จึงได้รับการอนุเคราะห์ค่าใช้จ่ายในการตรวจพังผืดทั้ง 3 วิธี ไม่ได้ขอรับทุนสนับสนุนจากแหล่งทุนอื่นๆ

วิธีการที่เกี่ยวข้องกับการวิจัย

ข้อมูลต่อไปนี้จะอธิบายถึงการศึกษาวิจัย ในกรณีที่ท่านตัดสินใจเข้าร่วมการวิจัย และได้ลงนามในหนังสือยินยอมเข้าร่วมการวิจัยฉบับนี้

1. ซักประวัติ ตรวจร่างกายและเก็บข้อมูลพื้นฐานโดยใช้แบบบันทึกข้อมูล และใช้แบบสอบถามเอสเอฟ-๓๖ (SF-๓๖) ฉบับภาษาไทยที่ได้รับการทดสอบประสิทธิภาพในการประเมินคุณภาพชีวิตของคนไทยมาแล้วก่อนหน้านี้ในการประเมินคุณภาพชีวิต โดยให้เวลาผู้เข้าร่วมวิจัยได้ตอบเองใช้เวลาเฉลี่ยในการทำแบบสอบถามประมาณ ๑๕ นาที หากผู้เข้าร่วมวิจัยอ่านหนังสือไม่ออกผู้วิจัยจะเป็นผู้ถามคำถามในแบบสอบถามให้ผู้ร่วมวิจัยตอบ
2. ผู้เข้าร่วมวิจัยจะได้รับการตรวจพังผืดตับทั้งสิ้น ๒ ครั้งห่างกัน ๑ ปี แต่ละครั้งจะได้รับการตรวจด้วยเครื่องมือ ๓ ชนิด ได้แก่
   a. ไฟโบรสแกน (Fibroscan บริษัทเอคโคเซนEchosens, ปารีส, ประเทศฝรั่งเศส)
   b. อัลตร้าซาวนด์ โดยใช้หลักการอาร์ฟี่ (ARFI-acoustic radiation force impulse imaging) โดยใช้เครื่องรุ่นโลจิกอีไนน์เอ็กดีเคลีย (LOGIQ E๙ Xdclear, บริษัทจีอี, วิสคอนซิน, สหรัฐอเมริกา)
   c. เครื่องตรวจคลื่นแม่เหล็กไฟฟ้า หรือเอ็มอาร์อี (MRE-Magnetic resonance elastography)
3. ผู้เข้าร่วมวิจัยจะได้รับการแนะนำเรื่องวิธีการเตรียมตัวให้งดน้ำและอาหารมาก่อนประมาณ ๒ ชั่วโมงก่อนทำการตรวจโดยเจ้าหน้าที่และนัดหมายวันและเวลาในการตรวจ

๔. ผู้ทำวิจัยจะขอเจาะเลือด เพื่อตรวจค่าการทำงานของตับและตรวจนับเม็ดเลือดหรือซีบีซี โดยเจาะเลือดทั้งสิ้น ๖ ซีซี

๕. เมื่อครบระยะเวลา ๑ ปีเจ้าหน้าที่จะโทรติดตามเพื่อให้ผู้เข้าร่วมวิจัยมาตรวจประเมินพังผืดตับโดยเครื่องมือทั้ง ๓ ชนิดอีกครั้ง

ความรับผิดชอบของอาสาสมัครผู้เข้าร่วมในโครงการวิจัย

เพื่อให้งานวิจัยนี้ประสบความสำเร็จ ผู้ทำวิจัยใคร่ขอความความร่วมมือจากท่าน โดยจะขอให้ท่านปฏิบัติตามคำแนะนำของผู้ทำวิจัยอย่างเคร่งครัด รวมทั้งแจ้งอาการผิดปกติต่าง ๆ ที่เกิดขึ้นกับท่านระหว่างที่ท่านเข้าร่วมในโครงการวิจัยให้ผู้ทำวิจัยได้รับทราบ

<u>ความเสี่ยงที่อาจได้รับ</u>

ผู้เข้าร่วมงานวิจัยนี้อาจเกิดความเสี่ยง เช่น เสียเวลา ไม่สะดวกสบายทั้งทางร่างกายและจิตใจสูญเสียรายได้

<u>ความเสี่ยงที่ได้รับจากการตรวจด้วยเครื่องมือ</u>

การตรวจพังผืดในตับโดยเครื่องมือทั้ง ๓ ชนิดได้แก่ เครื่องวัดความยืดหยุ่นของตับหรือไฟโบรสแกน อัลตร้าซาวนด์ เครื่องตรวจคลื่นแม่เหล็กไฟฟ้า อาจเกิดความเสี่ยงจากการตรวจต่อผู้เข้าร่วมวิจัย เช่นรู้สึกไม่สบาย อึดอัดจากการเข้าเครื่องตรวจ อาจเกิดมีอาการกลัวที่แคบขณะทำการถ่ายภาพด้วยเครื่องแม่เหล็กไฟฟ้า อาจมีความรู้สึกเจ็บเล็กน้อยจากการวางหัวตรวจบริเวณชายโครงด้านขวา

<u>ความเสี่ยงที่ได้รับจากการเจาะเลือด</u>

ท่านมีโอกาสที่จะเกิดอาการเจ็บ เลือดออก ช้ำจากการเจาะเลือด อาการบวมบริเวณที่เจาะเลือดหรือหน้ามืด และโอกาสที่จะเกิดการติดเชื้อบริเวณที่เจาะเลือดพบได้น้อยมาก และจะไม่มีผลกระทบต่อสุขภาพของท่าน จากการเข้าร่วมงานวิจัยนี้

<u>ความเสี่ยงที่ไม่ทราบแน่นอน</u>

ท่านอาจเกิดอาการข้างเคียง หรือความไม่สบาย นอกเหนือจากที่ได้แสดงในเอกสารฉบับนี้ ซึ่งอาการข้างเคียงเหล่านี้เป็นอาการที่ไม่เคยพบมาก่อน เพื่อความปลอดภัยของท่าน ควรแจ้งผู้ทำวิจัยให้ทราบทันทีเมื่อเกิดความผิดปกติใดๆ เกิดขึ้น

หากท่านมีข้อสงสัยใดๆ เกี่ยวกับความเสี่ยงที่อาจได้รับจากการเข้าร่วมในโครงการวิจัย ท่านสามารถสอบถามจากผู้ทำวิจัยได้ตลอดเวลา

หากมีการค้นพบข้อมูลใหม่ ๆ ที่อาจมีผลต่อความปลอดภัยของท่านในระหว่างที่ท่านเข้าร่วมในโครงการวิจัย ผู้ทำวิจัยจะแจ้งให้ท่านทราบทันที เพื่อให้ท่านตัดสินใจว่าจะอยู่ในโครงการวิจัยต่อไปหรือจะขอถอนตัวออกจากการวิจัย

<u>การพบแพทย์นอกตารางนัดหมายในกรณีที่เกิดอาการข้างเคียง</u>

หากมีอาการข้างเคียงใด ๆ เกิดขึ้นกับท่าน ขอให้ท่านรีบมาพบแพทย์ที่สถานพยาบาลทันที ถึงแม้ว่าจะอยู่นอกตารางการนัดหมาย เพื่อแพทย์จะได้ประเมินอาการข้างเคียงของท่าน และให้การรักษาที่เหมาะสมทันที หากอาการดังกล่าวเป็นผลจากการเข้าร่วมในโครงการวิจัย ท่านจะไม่เสียค่าใช้จ่าย

<u>ประโยชน์ที่อาจได้รับ</u>

ท่านอาจจะไม่ได้รับประโยชน์ใดๆ จากการเข้าร่วมในการวิจัยนี้ แต่จะทำให้ท่านได้ทราบปริมาณพังผืดในตับและทราบว่าเมื่อติดตามไป ๑ ปีพังผืดมีการเปลี่ยนแปลงอย่างไร อีกทั้งแต่ผลของการศึกษาวิจัยในครั้งนี้จะก่อประโยชน์ให้เกิดองค์ความรู้ใหม่ในการประยุกต์ใช้ไปสู่การดูแลรักษาผู้ป่วยโรคตับอักเสบเรื้อรังซึ่งมีอยู่เป็นจำนวนมากในภายภาคหน้า

<u>วิธีการและรูปแบบการรักษาอื่น ๆ ซึ่งมีอยู่สำหรับอาสาสมัคร</u>

ท่านไม่จำเป็นต้องเข้าร่วมโครงการวิจัยนี้เพื่อประโยชน์ในการรักษาโรคที่ท่านเป็นอยู่ เนื่องจากมีแนวทางการรักษาอื่น ๆ หลายแบบสำหรับรักษาโรคของท่านได้ ดังนั้นจึงควรปรึกษาแนวทางการรักษาวิธีอื่นๆ กับแพทย์ผู้ให้การรักษาท่านก่อนตัดสินใจเข้าร่วมในการวิจัย

<u>ข้อปฏิบัติของท่านขณะที่ร่วมในโครงการวิจัย</u>

ขอให้ท่านปฏิบัติดังนี้

- ขอให้ท่านให้ข้อมูลทางการแพทย์ของท่านทั้งในอดีต และปัจจุบัน แก่ผู้ทำวิจัยด้วยความสัตย์จริง
- ขอให้ท่านแจ้งให้ผู้ทำวิจัยทราบความผิดปกติที่เกิดขึ้นระหว่างที่ท่านร่วมในโครงการวิจัย

<u>อันตรายที่อาจเกิดขึ้นจากการเข้าร่วมในโครงการวิจัยและความรับผิดชอบของผู้ทำวิจัย/ผู้สนับสนุนการวิจัย</u>

หากพบอันตรายที่เกิดขึ้นจากการเข้าร่วมการวิจัย ท่านจะได้รับการรักษาอย่างเหมาะสมทันที หากท่านปฏิบัติตามคำแนะนำของทีมผู้ทำวิจัยแล้ว ผู้ทำวิจัย/ผู้สนับสนุนการวิจัยยินดีจะรับผิดชอบค่าใช้จ่ายในการรักษาพยาบาลของท่าน อีกทั้งจะได้รับการชดเชยการสูญเสียเวลา เสียรายได้ตามความเหมาะสม

ในกรณีที่ท่านได้รับอันตรายใด ๆ หรือต้องการข้อมูลเพิ่มเติมที่เกี่ยวข้องกับโครงการวิจัย ท่านสามารถติดต่อกับผู้ทำวิจัยได้ตลอด ๒๔ ชั่วโมง

<u>ค่าใช้จ่ายของท่านในการเข้าร่วมการวิจัย</u>

ค่าใช้จ่ายที่เกี่ยวข้องกับโครงการวิจัย เช่น ค่าธรรมเนียมในการตรวจด้วยเครื่องมือ และการตรวจเลือด ผู้วิจัยจะเป็นผู้รับผิดชอบทั้งหมด

<u>ค่าตอบแทนสำหรับผู้เข้าร่วมวิจัย</u> (ถ้าไม่มีให้ระบุว่าไม่มี)

ไม่มีเงินค่าตอบแทนจากการเข้าร่วมในการวิจัยรวมทั้งค่าเดินทางสำหรับผู้เข้าร่วมวิจัย

<u>การเข้าร่วมและการสิ้นสุดการเข้าร่วมโครงการวิจัย</u>

การเข้าร่วมในโครงการวิจัยครั้งนี้เป็นไปโดยความสมัครใจ หากท่านไม่สมัครใจจะเข้าร่วมการศึกษาแล้ว ท่านสามารถถอนตัวได้ตลอดเวลา การขอถอนตัวออกจากโครงการวิจัยจะไม่มีผลต่อการดูแลรักษาโรคของท่านแต่อย่างใด

ผู้ทำวิจัยอาจถอนท่านออกจากการเข้าร่วมการวิจัย เพื่อเหตุผลด้านความปลอดภัยของท่าน หรือเมื่อผู้สนับสนุนการวิจัยยุติการดำเนินงานวิจัย หรือ ในกรณีดังต่อไปนี้

- ท่านไม่สามารถปฏิบัติตามคำแนะนำของผู้ทำวิจัย
- ท่านตั้งครรภ์ระหว่างที่เข้าร่วมโครงการวิจัย
-

<u>การปกป้องรักษาข้อมูลความลับของอาสาสมัคร</u>

ข้อมูลที่อาจนำไปสู่การเปิดเผยตัวท่าน จะได้รับการปกปิดและจะไม่เปิดเผยแก่สาธารณชน ในกรณีที่ผลการวิจัยได้รับการตีพิมพ์ ชื่อและที่อยู่ของท่านจะต้องได้รับการปกปิดอยู่เสมอ โดยจะใช้เฉพาะรหัสประจำโครงการวิจัยของท่าน

จากการลงนามยินยอมของท่าน ผู้ทำวิจัย และผู้สนับสนุนการวิจัย คณะกรรมการจริยธรรมการวิจัย ผู้ตรวจสอบการวิจัย และหน่วยงานควบคุมระเบียบกฎหมาย สามารถเข้าไปตรวจสอบบันทึกข้อมูลทางการแพทย์ของ

ท่านได้แม้จะสิ้นสุดโครงการวิจัยแล้วก็ตาม โดยไม่ละเมิดสิทธิของท่านในการรักษาความลับเกินขอบเขตที่กฎหมายและระเบียบกฎหมายอนุญาตไว้

จากการลงนามยินยอมของท่าน แพทย์ผู้ทำวิจัยสามารถบอกรายละเอียดที่เกี่ยวกับการเข้าร่วมโครงการวิจัยนี้ของท่านให้แก่แพทย์ผู้รักษาท่านได้

การยกเลิกการให้ความยินยอม

หากท่านต้องการยกเลิกการให้ความยินยอมดังกล่าว ท่านสามารถแจ้ง หรือเขียนบันทึกขอยกเลิกการให้คำยินยอม โดยส่งไปที่ นายแพทย์ปิยะวัฒน์ โกมลมิศร์ สาขาวิชาโรคระบบทางเดินอาหารและตับ ภาควิชาอายุรศาสตร์ คณะแพทยศาสตร์ โรงพยาบาลจุฬาลงกรณ์ ตึกภูมิสิริฯ ชั้น ๒ (ฝั่งตึกเจริญสมศรี) เลขที่ ๑๘๗๓ ถนน พระรามที่ ๔ แขวงปทุมวัน เขตปทุมวัน กรุงเทพมหานคร ๑๐๓๓๐ โทร. ๐๒-๒๕๖-๔๒๖๕

หากท่านขอยกเลิกการให้คำยินยอมหลังจากที่ท่านได้เข้าร่วมโครงการวิจัยแล้ว ข้อมูลส่วนตัวของท่านจะไม่ถูกบันทึกเพิ่มเติม อย่างไรก็ตามข้อมูลอื่น ๆ ของท่านอาจถูกนำมาใช้เพื่อประเมินผลการวิจัย และท่านจะไม่สามารถกลับมาเข้าร่วมในโครงการนี้ได้อีก ทั้งนี้เนื่องจากข้อมูลของท่านที่จำเป็นสำหรับใช้เพื่อการวิจัยไม่ได้ถูกบันทึก

<u>การจัดการกับตัวอย่างชีวภาพที่เหลือ</u>

หลังจากเสร็จสิ้นงานวิจัย จะมีการเก็บตัวอย่างเลือดและ ดีเอ็นเอ ไว้ที่หน่วยโรคระบบทางเดินอาหาร สาขาวิชาโรคทางเดินอาหาร โรงพยาบาลจุฬาลงกรณ์ ถนนพระรามสี่ ปทุมวัน กรุงเทพ ๑๐๓๓๐ เพื่อใช้ในการตรวจหาความหลากหลายทางชีวภาพตัวอื่นในอนาคต โดยจะเก็บไว้นาน ๑๕ ปีจึงจะทำลาย ถ้ามีการนำตัวอย่างเหล่านี้มาใช้จะมีการเขียนเพื่อขอคำอนุญาตจากคณะกรรมการพิจารณาจริยธรรมการวิจัยก่อน

<u>สิทธิ์ของผู้เข้าร่วมในโครงการวิจัย</u>

ในฐานะที่ท่านเป็นผู้เข้าร่วมในโครงการวิจัย ท่านจะมีสิทธิ์ดังต่อไปนี้

๑. ท่านจะได้รับทราบถึงลักษณะและวัตถุประสงค์ของการวิจัยในครั้งนี้
๒. ท่านจะได้รับการอธิบายเกี่ยวกับระเบียบวิธีการของการวิจัยทางการแพทย์ รวมทั้งยาและอุปกรณ์ที่ใช้ในการวิจัยครั้งนี้
๓. ท่านจะได้รับการอธิบายถึงความเสี่ยงและความไม่สบายที่จะได้รับจากการวิจัย
๔. ท่านจะได้รับการอธิบายถึงประโยชน์ที่ท่านอาจจะได้รับจากการวิจัย
๕. ท่านจะได้รับการเปิดเผยถึงทางเลือกในการรักษาด้วยวิธีอื่น ยา หรืออุปกรณ์ซึ่งมีผลดีต่อท่านรวมทั้งประโยชน์และความเสี่ยงที่ท่านอาจได้รับ
๖. ท่านจะได้รับทราบแนวทางในการรักษา ในกรณีที่พบโรคแทรกซ้อนภายหลังการเข้าร่วมในโครงการวิจัย
๗. ท่านจะมีโอกาสได้ซักถามเกี่ยวกับงานวิจัยหรือขั้นตอนที่เกี่ยวข้องกับงานวิจัย
๘. ท่านจะได้รับทราบว่าการยินยอมเข้าร่วมในโครงการวิจัยนี้ ท่านสามารถขอถอนตัวจากโครงการเมื่อไรก็ได้ โดยผู้เข้าร่วมในโครงการวิจัยสามารถขอถอนตัวจากโครงการโดยไม่ได้รับผลกระทบใด ๆ ทั้งสิ้น
๙. ท่านจะได้รับเอกสารข้อมูลคำอธิบายสำหรับผู้เข้าร่วมในโครงการวิจัยและสำเนาเอกสารใบยินยอมที่มีทั้งลายเซ็นและวันที่
๑๐. ท่านมีสิทธิ์ในการตัดสินใจว่าจะเข้าร่วมในโครงการวิจัยหรือไม่ก็ได้ โดยปราศจากการใช้อิทธิพลบังคับข่มขู่หรือการหลอกลวง

หากท่านไม่ได้รับการชดเชยอันควรต่อการบาดเจ็บหรือเจ็บป่วยที่เกิดขึ้นโดยตรงจากการวิจัย หรือท่านไม่ได้รับการปฏิบัติตามที่ปรากฏในเอกสารข้อมูลคำอธิบายสำหรับผู้เข้าร่วมในการวิจัย ท่านสามารถร้องเรียนได้ที่สำนักงาน**คณะกรรมการจริยธรรมการวิจัย คณะแพทยศาสตร์ จุฬาลงกรณ์มหาวิทยาลัย ตึกอานันทมหิดลชั้น 3 โรงพยาบาลจุฬาลงกรณ์ ถนนพระราม 4 ปทุมวัน กรุงเทพฯ 10330 โทร 0-2256-4493 ในเวลาราชการ**

การลงนามในเอกสารให้ความยินยอม ไม่ได้หมายความว่าท่านได้สละสิทธิ์ทางกฎหมายตามปกติที่ท่านพึงมี

ขอขอบคุณในการให้ความร่วมมือของท่านมา ณ ที่นี้

....

การวิจัยเรื่อง การเปรียบเทียบประสิทธิภาพของเครื่องวัดความยืดหยุ่นของตับ อัลตร้าซาวนด์ เครื่องตรวจคลื่นแม่เหล็กไฟฟ้าในการประเมินภาวะพังผืดตับในผู้ป่วยไวรัสตับอักเสบซีและตับคั่งไขมันที่ไม่ได้เกิดจากแอลกอฮอล์ และการเปลี่ยนแปลงของพังผืดตับติดตามไป 1 ปี

วันให้คำยินยอม วันที่.............เดือน.......................................พ.ศ...

ข้าพเจ้า นาย/นาง/นางสาว...ที่อยู่ ....ได้อ่านรายละเอียดจากเอกสารข้อมูลสำหรับผู้เข้าร่วมโครงการวิจัยวิจัยที่แนบมาฉบับวันที่.................................. และข้าพเจ้ายินยอมเข้าร่วมโครงการวิจัยโดยสมัครใจ

ข้าพเจ้าได้รับสำเนาเอกสารแสดงความยินยอมเข้าร่วมในโครงการวิจัยที่ข้าพเจ้าได้ลงนาม และ วันที่ พร้อมด้วยเอกสารข้อมูลสำหรับผู้เข้าร่วมโครงการวิจัย ทั้งนี้ก่อนที่จะลงนามในใบยินยอมให้ทำการวิจัยนี้ ข้าพเจ้าได้รับการอธิบายจากผู้วิจัยถึงวัตถุประสงค์ของการวิจัย ระยะเวลาของการทำวิจัย วิธีการวิจัย อันตราย หรืออาการที่อาจเกิดขึ้นจากการวิจัย รวมทั้งประโยชน์ที่จะเกิดขึ้นจากการวิจัย และแนวทางรักษาโดยวิธีอื่นอย่างละเอียด ข้าพเจ้ามีเวลาและโอกาสเพียงพอในการซักถามข้อสงสัยจนมีความเข้าใจอย่างดีแล้ว โดยผู้วิจัยได้ตอบคำถามต่าง ๆ ด้วยความเต็มใจไม่ปิดบังซ่อนเร้นจนข้าพเจ้าพอใจ

ข้าพเจ้ารับทราบจากผู้วิจัยว่าหากเกิดอันตรายใด ๆ จากการวิจัยดังกล่าว ข้าพเจ้าจะได้รับการรักษาพยาบาลโดยไม่เสียค่าใช้จ่าย และไม่ได้รับเงินจากผู้สนับสนุนการวิจัย

ข้าพเจ้ามีสิทธิที่จะบอกเลิกเข้าร่วมในโครงการวิจัยเมื่อใดก็ได้ โดยไม่จำเป็นต้องแจ้งเหตุผล และการบอกเลิกการเข้าร่วมการวิจัยนี้ จะไม่มีผลต่อการรักษาโรคหรือสิทธิอื่น ๆ ที่ข้าพเจ้าจะพึงได้รับต่อไป

ผู้วิจัยรับรองว่าจะเก็บข้อมูลส่วนตัวของข้าพเจ้าเป็นความลับ และจะเปิดเผยได้เฉพาะเมื่อได้รับการยินยอมจากข้าพเจ้าเท่านั้น บุคคลอื่นในนามของบริษัทผู้สนับสนุนการวิจัย คณะกรรมการพิจารณาจริยธรรมการวิจัยในคน อาจได้รับอนุญาตให้เข้ามาตรวจและประมวลข้อมูลของข้าพเจ้า ทั้งนี้จะต้องกระทำไปเพื่อวัตถุประสงค์เพื่อตรวจสอบความถูกต้องของข้อมูลเท่านั้น โดยการตกลงที่จะเข้าร่วมการศึกษานี้ข้าพเจ้าได้ให้คำยินยอมที่จะให้มีการตรวจสอบข้อมูลประวัติทางการแพทย์ของข้าพเจ้าได้

ผู้วิจัยรับรองว่าจะไม่มีการเก็บข้อมูลใด ๆ ของข้าพเจ้า เพิ่มเติม หลังจากที่ข้าพเจ้าขอยกเลิกการเข้าร่วมโครงการวิจัยและต้องการให้ทำลายเอกสารและ/หรือ ตัวอย่างที่ใช้ตรวจสอบทั้งหมดที่สามารถสืบค้นถึงตัวข้าพเจ้าได้

หลังจากเสร็จสิ้นงานวิจัย จะมีการเก็บตัวอย่างเลือดและ DNA ไว้ที่หน่วยโรคระบบทางเดินอาหาร เลขที่ ๑๘๗๓ ตึกภูมิสิริฯ ชั้น ๒ สาขาวิชาโรคทางเดินอาหาร โรงพยาบาลจุฬาลงกรณ์ ถนนพระรามสี่ ปทุมวัน กรุงเทพ ๑๐๓๓๐ เพื่อใช้ในการ

ตรวจหาความหลากหลายทางชีวภาพตัวอื่นในอนาคต โดยจะเก็บไว้นาน ๑๕ ปีจึงจะทำลาย ถ้ามีการนำตัวอย่างเหล่านี้มาใช้จะมีการเขียนเพื่อขอคำอนุญาตจากคณะกรรมการพิจารณาจริยธรรมการวิจัยก่อน

ข้าพเจ้าจะ ☐ ยินยอม ☐ ไม่ยินยอม

ข้าพเจ้าเข้าใจว่า ข้าพเจ้ามีสิทธิ์ที่จะตรวจสอบหรือแก้ไขข้อมูลส่วนตัวของข้าพเจ้าและสามารถยกเลิกการให้สิทธิในการใช้ข้อมูลส่วนตัวของข้าพเจ้าได้ โดยต้องแจ้งให้ผู้วิจัยรับทราบ

ข้าพเจ้าได้ตระหนักว่าข้อมูลในการวิจัยรวมถึงข้อมูลทางการแพทย์ของข้าพเจ้าที่ไม่มีการเปิดเผยชื่อ จะผ่านกระบวนการต่าง ๆ เช่น การเก็บข้อมูล การบันทึกข้อมูลในแบบบันทึกและในคอมพิวเตอร์ การตรวจสอบ การวิเคราะห์ และการรายงานข้อมูลเพื่อวัตถุประสงค์ทางวิชาการ รวมทั้งการใช้ข้อมูลทางการแพทย์ในอนาคตหรือการวิจัยทางด้านเภสัชภัณฑ์เท่านั้น

ข้าพเจ้าได้อ่านข้อความข้างต้นและมีความเข้าใจดีทุกประการแล้ว ยินดีเข้าร่วมในการวิจัยด้วยความเต็มใจ จึงได้ลงนามในเอกสารแสดงความยินยอมนี้

....ลงนามผู้ให้ความยินยอม

(...) ชื่อผู้ยินยอมตัวบรรจง

วันที่ ....เดือน...................................พ.ศ............................

ข้าพเจ้าได้อธิบายถึงวัตถุประสงค์ของการวิจัย วิธีการวิจัย อันตราย หรืออาการไม่พึงประสงค์หรือความเสี่ยงที่อาจเกิดขึ้นจากการวิจัย หรือจากยาที่ใช้ รวมทั้งประโยชน์ที่จะเกิดขึ้นจากการวิจัยอย่างละเอียด ให้ข้าพเจ้าได้ทราบและมีความเข้าใจดีแล้ว พร้อมลงนามลงในเอกสารแสดงความยินยอมด้วยความเต็มใจ

....ลงนามผู้ทำวิจัย

(...) ชื่อผู้ทำวิจัย ตัวบรรจง

วันที่ ....เดือน...................................พ.ศ............................

....ลงนามพยาน

(...) ชื่อพยาน ตัวบรรจง

วันที่ ....เดือน...................................พ.ศ............................
