# Supplementary material for: Changes in hepatic fibrosis and vitamin D levels after viral hepatitis C eradication using direct-acting antiviral therapy
Source: BMC Gastroenterol. 2020 Oct 17;20:346. doi: 10.1186/s12876-020-01485-8 (PMC7568415; doi:10.1186/s12876-020-01485-8)
Supplement: Supplementary file 2 — Additional file 2: Supplementary data 2: Study protocol (English version). [file 12876_2020_1485_MOESM2_ESM.pdf]

SUBMISSION PROPOSAL FOR
INSTITUTIONAL REVIEW BOARD
FACULTY OF MEDICINE, CHULALONGKORN UNIVERSITY
BANGKOK, THAILAND

Table of contents	Page
Front page	2
1. Background	3
2. Hypothesis and research questions	5
3. Research design and methodology	6
4. References	10
5. Appendix	
5.1 Case record form	12
5.2 Patient information sheet	14
5.3 Informed consent sheet	20

RESEARCH PROPOSAL SUBMITTED FOR
INSTITUTIONAL REVIEW BOARD
FACULTY OF MEDICINE, CHULALONGKORN UNIVERSITY
BANGKOK, THAILAND

Title: Comparison of transient elastography, acoustic radiation force impulse and magnetic resonance imaging for non-invasive staging of liver fibrosis in patients with chronic hepatitis C and nonalcoholic fatty liver disease: 1-year follow-up of fibrosis progression

Principle investigators:

1. Dr. Piyawat Komolmit

Affiliation:

Division of Gastroenterology and Hepatology

Department of Medicine,

Faculty of Medicine Chulalongkorn University

1. Background

Chronic hepatitis C and non-alcoholic liver disease (NAFLD) are the leading causes of chronic hepatitis worldwide. The persistent inflammation of liver parenchyma results in progressive fibrosis, which ultimately turns to liver cirrhosis. Liver biopsy is the gold standard method for fibrosis evaluation. However, it is not free of risk. Bleeding complication was reported with incidence of 1:2,500 to 1:10,000 and fatal complication was also reported about 0.09%. Nowadays, there are new devices and technology for evaluating liver fibrosis instead of invasive liver biopsy, which help us to detect an early stage of liver fibrosis.

Ultrasound elastography and magnetic resonance imaging were developed in recent years. Unfortunately, there was no data in Thai chronic hepatitis patients. Therefore, we conducted this study to evaluate the performance of ultrasound elastography and magnetic resonance imaging in Thai patients.

Chronic hepatitis C is the leading problems among world population. Recent study reports more than 185 million people suffering from hepatitis C virus (HCV) infection (1). Non-alcoholic fatty liver disease (NAFLD) is another concerning health issue, especially in the western countries. Incidence of NAFLD has been reported about 19-46% (2). Both hepatitis C and NAFLD are the major causes of chronic liver disease, hepatocellular carcinoma in Thailand.

Definition of liver fibrosis and diagnosis

Hepatic fibrosis occurs after the event of liver injury. Hepatic stellate cells (HSCs) are activated and transformed to activated HSCs or myofibroblasts. This active form of HSCs control a fibrogenic cytokines eg. transforming growth factor (TGF-β), platelet derived growth factor (PDGF), connective tissue growth factor (CTGF) leading to accumulation of extracellular matrix and eventually turning to liver fibrosis (3,4). Nowadays, we believe that the regression of liver fibrosis can occur after the resolution of liver injury.

Classification of liver fibrosis can predict the severity and prognosis of liver diseases. Liver biopsy is a standard method for determining the definite fibrosis. Many purposed classifications are used in clinical practice such as METAVIR, Knodell, Schuer, Ishak score (5). Moreover, these classified data are correlated with clinical presentation and prognosis of chronic hepatitis patients.

Table 1. Liver fibrosis classification

**METAVIR score**	
**Stage**	
**0**	No fibrosis
**1**	Mild fibrosis-portal fibrosis
**2**	Moderate fibrosis-portal fibrosis and few septa
**3**	Severe fibrosis-numerous septa without cirrhosis
**4**	Cirrhosis
**ISHAK score**	
**Stage**	
**0**	No fibrosis
**1**	Fibrous expansion of **some** portal area ± short fibrous septa
**2**	Fibrous expansion of **most** portal area ± short fibrous septa
**3**	Fibrous expansion of some portal area with occasional portal to portal bridging
**4**	Fibrous expansion of some portal area with marked bridging as well as portal to central
**5**	Marked bridging (portal to portal and/or portal to central) with occasional nodules
**6**	Cirrhosis, probable or definite

However, liver biopsy is not free of risk. Many complications have been reported; pain 84%, bleeding 1:2500-1:10000, death <1:10000. Other possible complications are pneumo/hemothorax, viscous organs perforation, bile peritonitis, bacteremia, sepsis, hemobilia, neuralgia and ventricular arrthymia (6). Moreover, sampling error and intraobserver variations can lead to inaccurate interpretation (7). Thus, nowadays, many non-invasive devices have been invented to use instead of conventional liver biopsy.

Non-invasive tests to assess staging of liver fibrosis

1. **Serum biomarkers:** based on biomarkers from fibrotic pathways eg. Fibrotest® (Biopredictive, Paris, France), AST to Platelet Ratio (APRI), Fibrospect® (Promotheus Laboratory Inc, San Diego, USA), Enhanced Liver Fibrosis score® (ELF) (Siemens Healthcare,

Erlangen, Germany). Several data have been approved these biomarkers for liver fibrosis assessment (8).

2. **Liver stiffness measurement (liver elastography):** based on elasticity of the liver
    a. Static or quasistatic (stain imaging) eg. eMode (Hitachi), eSie Touch (Siemens Healthcare), SonixTOUCH (Ultrasonix), Strain-based elastography (Philips Healthcare), Strain elastography (GE healthcare)
    b. Dynamic (Shear-wave imaging)
        i. Transient (Ultrasound-based elastography)
            1. One-dimensional Transient elastrography: Fibroscan (Echosens)
            2. Point shear wave elastography (pSWE) or acoustic radiation force impulse imaging (ARFI) ได้แก่ Virtual touch tissue quantification™, (Siemens Healthcare), elastography point quantification (ElastPQ™, Philips), shear wave elastography (GE healthcare)
            3. Two-dimensional shear wave elastography (2D-SWE) eg. Aixplorer™ (Supersonic Imagine, France)
        ii. Continuous eg. Magnetic resonance elastography (MRE) (GE healthcare, Philips Healthcare, Siemens Healthcare)

2. Objectives

Primary endpoint

To compare the performance of fibroscan, ultrasound elastography and magnetic resonance imaging elastography in chronic hepatitis patients

Secondary endpoint

1. To study the change of liver fibrosis within 1 years, measured by fibroscan, ultrasound elastography and magnetic resonance imaging elastography in Thai chronic hepatitis patients
2. To study the quality of life in Thai chronic hepatitis patients

3. Hypothesis

Primary research question

What are the differences among fibroscan, ultrasound elastography and magnetic resonance imaging elastography?

Secondary research question

1. How is the change in liver fibrosis in chronic hepatitis C and NAFLD patients in 1-year period?
2. How is the quality of life in chronic hepatitis C and NAFLD patients?

4. Key words

Chronic hepatitis C infection, non-alcoholic fatty liver diease, Elastography

5. Research design

Prospective, analytic study

6. Research methodology

Target population

Chronic hepatitis C and NAFLD patients

Inclusion criteria

1. Patients diagnosed with chronic hepatitis C (positive anti-HCV antibody more than 6 months)
2. Age > 18 years

Exclusion criteria

1. Alcohol drinking >14 units/week in male, or >7 units/week in female
2. HIV coinfection
3. Hepatitis B (HBV) coinfection
4. Decline to participate in the study
5. Other chronic hepatitis from other causes eg, autoimmune hepatits, hemochromatosis, Wilson disease, cholestatic or vascular liver diaseases
6. Decompensated cirrhosis
7. Contraindication for MRE; claustrophobia, metallic implant, pregnancy

Research protocol

1. Inform objectives, benefit and risk and get a formal informed consent from the participants
2. History taking, physical examination and data collection

3. Blood taking in clotted tube and EDTA tube (6 mL)
4. Leave specimen in room-temperature for 30 minutes
5. Centrifuge the specimen for 15 minutes
6. Serum collection at -70°C refrigerator
7. Perform liver fibrosis assessment by 3 methods; Fibroscan, ultrasound elastography and MRE

How to manage the residual biologic material

After the study ends, your residual biologic material; blood, will be collected at Division of gastroenterology, Chulalongkorn hospital, Rama IV road, Pathumwan, Bangkok 10330 for evaluate various of biologic markers in the future. We will collect your blood for 15 years and then the remaining specimen will be destroyed after that. If we would like to take any of your collected specimen, we will ask for a permission to Ethic Committee (EC) before using the biologic specimen.

Sample size calculation

$$\text{Number} = \frac{[Z_{\alpha/2} + Z_{\beta}]^2 \sigma^2}{(d)^2}$$

$\alpha = 0.05$ → $Z_{\alpha/2} = 1.96$

$\beta = 0.1$ → $Z_{\beta} = 1.28$

σ^2 = variance of difference $= SD1^2 + SD2^2 - 2(r)SD1SD2$

d = mean of difference = (X1 – X2)

From previous HCV data

X1 = the mean transient elastogram to detect advanced fibrosis from Fibroscan = 7.1

X2 = the mean transient elastogram to detect advanced fibrosis from MRE = 4.15

SD1 = SD of liver fibrosis measured by Fibroscan = 0.54

SD1 = SD of liver fibrosis measured by MRE = 8.2

r = Correlation between TE และ MRE = 0

$\sigma^2 = (0.54)^2 + (8.2)^2 - 2(0)(0.54)(8.2) = 67.5$

$$\text{Calculated sample size} = \frac{[1.96 + 1.28]^2\ 67.5}{(7.1 - 4.15)^2} = 81.4$$

Estimated 20% drop-out rate. Therefore, number of chronic hepatitis patients needed = 98 patients

7. Data collection

Data will be collected by investigators and research team, Division of gastroenterology, Chulalongkorn hospital

8. Data analysis and statistics

Qualitative or categorical data will be presented as frequency, percentage as appropriate. Quatitative data will be presented as mean, median and standard deviation (SD.)

Repated ANOVA is used to analyse the difference among three devices. P-value < 0.05 defines as statistical significance.

MRE is recognized as a gold standard in this study instead of liver biopsy

9. Expected or anticipated benefit gain

To add on the clinical information in liver fibrosis among Thai chronic hepatitis patients, which lead to appropriate management and care. The participants will also know the dynamic change in liver fibrosis within 1 year.

10. Challenges

Specimen collection problem and blood taking

11. Risk and investigator's responsibility

If the participants experience some possible complications during the trial, they will receive immediate treatment. Our investigator team will responsible for the cost of treatment.

If the participants experience any adverse events, they can contact the principle investigators by phone any time.

12. Timeline

12 months

13. Venue of the study

Hepatology clinic, Division of gastroenterology, Chulalongkorn university

14. Tabulation of Research Activities and Timeline

List	2016							2017			
	06	07	08	09	10	11	12	01	02	03	04
IRB process	■	■	■	■	■	■					
Specimen collection							■	■			
Assessment serum AFP, AFP-L3, PIVKAII									■		
Data collection									■	■	
Analysis									■	■	
Summary									■	■	
Manuscript and send for publications											■

15. Budget

List	Description	Total (Baht)
1. Wage		
1.1 Scientists	15,000 baht x 2	30,000
	**Total**	**30,000**
2. Expense		
2.1 Data and specimen collection	300 baht x 196 cases	58,800
	**Total**	**58,800**
3. Research officer		
3.1 Research assistant	2000 baht x 12 months	24,000
	**Total**	**24,000**
4. Others		
4.1 Office		5,000
4.2 Computer		10,000
4.3. Scientific methods		
1. buffy coat and serum	196 cases x 200 baht	39,200
2. DNA extraction	196 cases x 200 baht	39,200
3. SNPs analysis	196 cases x 400 baht	78,400
	**Total**	**156,800**
**TOTAL**		**268,800**

16. References

1. Mohd Hanafiah K, Groeger J, Flaxman AD, Wiersma ST. Global epidemiology of hepatitis C virus infection: new estimates of age-specific antibody to HCV seroprevalence. Hepatology 2013;57:1333-1342.

2. Williams CD, Stengel J, Asike MI, Torres DM, Shaw J, Contreras M, Landt CL, et al. Prevalence of nonalcoholic fatty liver disease and nonalcoholic steatohepatitis among a largely middle-aged population utilizing ultrasound and liver biopsy: a prospective study. Gastroenterology 2011;140:124-131.

3. Lee YA, Wallace MC, Friedman SL. Pathobiology of liver fibrosis: a translational success story. Gut 2015;64:830-841.

4. Urban ML, Manenti L, Vaglio A. Fibrosis--A Common Pathway to Organ Injury and Failure. N Engl J Med 2015;373:95-96.

5. Bedossa P, Poynard T. An algorithm for the grading of activity in chronic hepatitis C. The METAVIR Cooperative Study Group. Hepatology 1996;24:289-293.

6. Rockey DC, Caldwell SH, Goodman ZD, Nelson RC, Smith AD. Liver biopsy. Hepatology 2009;49:1017-1044.

7. Rousselet MC, Michalak S, Dupre F, Croue A, Bedossa P, Saint-Andre JP, Cales P. Sources of variability in histological scoring of chronic viral hepatitis. Hepatology 2005;41:257-264.

8. Fontana RJ, Goodman ZD, Dienstag JL, Bonkovsky HL, Naishadham D, Sterling RK, Su GL, et al. Relationship of serum fibrosis markers with liver fibrosis stage and collagen content in patients with advanced chronic hepatitis C. Hepatology 2008;47:789-798.

9. Kim SU, Choi GH, Han WK, Kim BK, Park JY, Kim DY, Choi JS, et al. What are 'true normal' liver stiffness values using FibroScan?: a prospective study in healthy living liver and kidney donors in South Korea. Liver Int 2010;30:268-274.

10. Castera L, Vergniol J, Foucher J, Le Bail B, Chanteloup E, Haaser M, Darriet M, et al. Prospective comparison of transient elastography, Fibrotest, APRI, and liver biopsy for the assessment of fibrosis in chronic hepatitis C. Gastroenterology 2005;128:343-350.

11. Zarski JP, Sturm N, Guechot J, Paris A, Zafrani ES, Asselah T, Boisson RC, et al. Comparison of nine blood tests and transient elastography for liver fibrosis in chronic hepatitis C: the ANRS HCEP-23 study. J Hepatol 2012;56:55-62.

12. Wong VW, Vergniol J, Wong GL, Foucher J, Chan HL, Le Bail B, Choi PC, et al. Diagnosis of fibrosis and cirrhosis using liver stiffness measurement in nonalcoholic fatty liver disease. Hepatology 2010;51:454-462.

13. Sporea I, Bota S, Peck-Radosavljevic M, Sirli R, Tanaka H, Iijima H, Badea R, et al. Acoustic Radiation Force Impulse elastography for fibrosis evaluation in patients with chronic hepatitis C: an international multicenter study. Eur J Radiol 2012;81:4112-4118.

14. Liu H, Fu J, Hong R, Liu L, Li F. Acoustic Radiation Force Impulse Elastography for the Non-Invasive Evaluation of Hepatic Fibrosis in Non-Alcoholic Fatty Liver Disease Patients: A Systematic Review & Meta-Analysis. PLoS One 2015;10:e0127782.

15. Fierbinteanu Braticevici C, Sporea I, Panaitescu E, Tribus L. Value of acoustic radiation force impulse imaging elastography for non-invasive evaluation of patients with nonalcoholic fatty liver disease. Ultrasound Med Biol 2013;39:1942-1950.

16. Shi Y, Xia F, Li QJ, Li JH, Yu B, Li Y, An H, et al. Magnetic Resonance Elastography for the Evaluation of Liver Fibrosis in Chronic Hepatitis B and C by Using Both Gradient-Recalled Echo and Spin-Echo Echo Planar Imaging: A Prospective Study. Am J Gastroenterol 2016.

17. Wu WP, Chou CT, Chen RC, Lee CW, Lee KW, Wu HK. Non-Invasive Evaluation of Hepatic Fibrosis: The Diagnostic Performance of Magnetic Resonance Elastography in Patients with Viral Hepatitis B or C. PLoS One 2015;10:e0140068.

18. Kishore R, Loomba R, Sterling RK. Editorial: A New Spin on Magnetic Resonance Elastography. Am J Gastroenterol 2016;111:834-837.

19. Shiha G, Ibrahim A, Helmy A, Sarin SK, Omata M, Kumar A, Bernstien D, et al. Asian-Pacific Association for the Study of the Liver (APASL) consensus guidelines on invasive and non-invasive assessment of hepatic fibrosis: a 2016 update. Hepatol Int 2016.

20. Loomba R, Cui J, Wolfson T, Haufe W, Hooker J, Szeverenyi N, Ang B, et al. Novel 3D Magnetic Resonance Elastography for the Noninvasive Diagnosis of Advanced Fibrosis in NAFLD: A Prospective Study. Am J Gastroenterol 2016;111:986-994.

# Case record form<br><br>Center of excellence in liver diseases,<br>King Chulalongkorn Memorial Hospital,<br>Thai Red Cross, Bangkok, Thailand	Patient code: ☐☐☐<br>Date of data record: (Day-Month-Year)<br>☐☐-☐☐-☐☐☐☐

Part 1: Basic information

1. Sex ☐1. Male ☐ 2. Female 2. Birth date ______ / ________ / _____________
3. Tel : ________- _________________ Mobile: ________- _________________
4. Address____
5. Hometown_______________
6. Body weight___________ kg.
7. Height____________________ cm.
8. Waist circumference __________ cm.
9. Underlying diseases
   - ☐ 1.N/A ☐ 2. None
   - ☐ 3. Yes
     - ☐ 3.1. CVS ☐ 3.2 CKD ☐ 3.3 Dyslipidemia
     - ☐ 3.4 DM ☐ 3.5 HT ☐ 3.6 อื่นๆ (ระบุ)___________________
10. Alcohol ☐ 1.Yes ☐ 2 .Quit > 6 months ☐ 3 .No ☐9. N/A
11. Blood trnsfusion ☐ 1. Yes, ______ ☐ 2. No ☐9. N/A
12. Tattoo ☐ 1. Yes, ______ ☐ 2. No ☐9. N/A
13. IVDU ☐ 1. Yes, ______ ☐ 2. No ☐9. N/A
14. Family history of hepatitis/HCC ☐ 1. Yes, ______ ☐ 2. No ☐9. N/A

Part 2: Diagnosis

15 .Etiology Dx date: _______________

☐ 1.HCV genotype ☐ 1. G1 ☐ 2. G 2 ☐ 3. G 3 ☐ 4. G 6 ☐5.อื่นๆระบุ____ ☐9.ไม่มีข้อมูล

Previous Rx ☐ 1. None ☐ 2. Yes _________________________

☐ 2.NAFLD

Previous Rx ☐ 1. None ☐ 2. Yes _________________________

16. Stage of disease ☐ 1. Chronic hepatitis ☐ 2. Cirrhosis ☐ 3. Not assess
17. Fibroscan Date________________ ☐ 1. CAP__________dB/m ☐ 2. TE______________Kpa
18. HCC ☐ 1 Yes ☐ 2. No

Part 3: Specimen collection

Date of collected specimen ☐☐ - ☐☐ - ☐☐☐☐

19. CBC: Hb________ Hct ________ WBC________ (%neu______) Plt ________ INR ________ PT________

20. Liver function tests: TB________ DB ________ AST ________ ALT ________ ALP________ Alb ________

Glb________

21. BUN ________ Cr________

22. FBS_______________ HbA1C_______________ mg%

TG_______________ LDL_______________ CHol_______________ HDL_______________

23. Viral hepatitis

1. Anti-HCV ☐ 1. Positive ☐ 2. Negative ☐ 9.N/A

2. HCV-viral load ☐ 1. Yes ☐☐ , ☐☐☐ , ☐☐☐ (IU/ml)

☐ 2. No ☐ 9. N/A

3. HCV Genotype ☐ 1.Yes ☐ 2. No ☐ 9. N/A

3.1 ☐ 1. G1 ☐ 2. G 2 ☐ 3. G 3 ☐ 4. G 6 ☐ 5.Other:____ ☐ 9.N/A

4. HBsAg ☐ 1. Positive ☐ 2. Negative ☐ 9.N/A

5. Anti-HIV ☐ 1. Positive ☐ 2. Negative ☐ 9.N/A

Part 4: Fibrosis assessment

24. Fibroscan Date________________ Probe ☐ 1. M ☐ 2. XL Success rate________% IQR_________%

☐ 1. CAP__________dB/m ☐ 2. TE______________Kpa

25. USG Date________________ ☐ 1. TE______________Kpa Success ☐ 1. Yes ☐ 2. No

26. MRE Date________________ ☐ 1. TE______________Kpa Success ☐ 1. Yes ☐ 2. No

Part 5: One-year follow up

Date of collected specimen ☐☐ - ☐☐ - ☐☐☐☐

27. CBC: Hb________ Hct ________ WBC________ (%neu______) Plt ________ INR ________ PT________

28. Liver function tests: TB________ DB ________ AST ________ ALT ________ ALP________ Alb ________

Glb________

29. BUN ________ Cr________

30. FBS_______________ HbA1C_______________ mg%

TG_______________ LDL_______________ CHol_______________ HDL_______________

31. Fibroscan Date________________ Probe ☐ 1. M ☐ 2. XL Success rate________% IQR_________%

☐ 1. CAP__________dB/m ☐ 2. TE______________Kpa

32. USG Date________________ ☐ 1. TE______________Kpa Success ☐ 1. Yes ☐ 2. No

33. MRE Date________________ ☐ 1. TE______________Kpa Success ☐ 1. Yes ☐ 2. No

34. HCV Treatment ☐ 1. No treatment

☐ 2. Treatment regimen______ duration ______

☐ 1. Cured ☐ 2. Not cured

Information for participants

Title of research: Comparison of transient elastography, acoustic radiation force impulse and magnetic resonance imaging for non-invasive staging of liver fibrosis in patients with chronic hepatitis C and nonalcoholic fatty liver disease: 1-year follow-up of fibrosis progression

Principle investigators:

1. Dr. Piyawat Komolmit

Affiliation:

Division of Gastroenterology and Hepatology Department of Medicine, Faculty of Medicine Chulalongkorn University

Tel. 02-2564265, 090-6764824

To participants

You are invited to participate in this clinical trial as you have chronic hepatitis.

Before your agreement to involve in this trial, please read this document thoroughly for your understanding of the reason of the research and the detail of the protocol. If you have any questions, please ask our investigator team or principle investigators.

You can ask for advice from your family, friend or your general practitioner. You have time for your decision freely. If you decide to participate in this trial, please sign the agreement at the end of this document.

Background

Chronic hepatitis C and non-alcoholic liver disease (NAFLD) are the leading causes of chronic hepatitis worldwide. The persistent inflammation of liver parenchyma results in progressive fibrosis, which ultimately turns to liver cirrhosis. Liver biopsy is the gold standard method for fibrosis evaluation. However, it is not free of risk. Bleeding complication was reported with incidence of 1:2,500 to 1:10,000 and fatal complication was also reported about 0.09%. Nowadays, there are new devices and technology for evaluating liver fibrosis instead of invasive liver biopsy, which help us to detect an early stage of liver fibrosis.

Ultrasound elastography and magnetic resonance imaging were developed in recent years. Unfortunately, there was no data in Thai chronic hepatitis patients. Therefore, we conducted this

study to evaluate the performance of ultrasound elastography and magnetic resonance imaging in Thai patients.

Objective of the study

Primary objective

To compare the performance of fibroscan, ultrasound elastography and magnetic resonance imaging elastography in chronic hepatitis patients

Secondary outcome

3. To study the change of liver fibrosis within 1 years, measured by fibroscan, ultrasound elastography and magnetic resonance imaging elastography in Thai chronic hepatitis patients
4. To study the quality of life in Thai chronic hepatitis patients

Number of participants: 80 persons

Methods involve in this trial

After your agreement to participate in this trial, our investor team would like to the following tests.

1. History taking, physical examination, and evaluating quality of life by SF-36, which already validated in Thai population.
2. Liver fibrosis evaluation two times within 1 year by 3 special devices
    a. Fibroscan (Echosens, Paris, France)
    b. Ultrasound elastography (ARFI-acoustic radiation force impulse imaging) by LOGIQ E9 Xdclear, GE, Wisconsin, USA)
    c. MRE-Magnetic resonance elastography
3. Participant should fast for 2-hour duration before fibrosis evaluation.
4. Our investigator will take your blood (6 ml) to evaluate liver function, complete blood count and other related laboratory data.
5. Within 1 year, our investigator will make an appointment for re-evaluate liver fibrosis with three special devices and blood test.

Responsibility of the participants

For success of this study, we would like to ask participants to have discipline to comply with the protocol. If you have any abnormal symptoms during this study, please contact the investigator team.

Risk of the study

This study may possibly interfere personal daily life. You may lose your time and feel physically and mentally uncomfortable during the study protocol.

Risk of the special devices

The special device, including Fibroscan, Ultrasound elastography and MRE-Magnetic resonance elastography, may cause some uncomfortable feeling during the test protocol. Some participants may experience the clustophobic sensation and pain at right costal area during the test.

Risk from drawing blood sample

You may experience of pain at the puncture site, minor bleeding, ecchymosis, edema, syncope and local infection, which rarely happen.

Risk from other things

You might experience of some other symptoms not mention in this document as not seen before. For your safety, please report of any symptoms you may concern to the investigator team at any time.

If there is any new reports of any safety concern regarding the medications used in this trial, we will inform all participants as soon as possible and you may decide to continue or pull out from the study.

How to see the doctor for your concern of any adverse events

You can contact the principle investigators or the team at any time in case you experience of some symptoms or concern of any adverse events. Immediate advice or treatment will be provided.

Benefit from the study

To participate in this trial, you might know the dynamic changes of fibrosis in your liver, which could lead to an appropriate treatment and follow-up. However, this will not be a guarantee.

Other methods or managements for the participants

You have no need to be in this clinical trial for expecting of the treatment. As there might be other ways of treatment for your disease. You may ask the doctor or your GP before making decision to participate in this clinical trial.

Practical points for participants during the trial

Please read carefully

- Please give your information regarding your health and history of diseases or treatment.
- Please inform our team if you experience any symptoms of concern.

Adverse events or complications happened during the trial and responsibility

If you have any complications during the trial, you will receive immediate treatment. Our investigator team will responsible for the cost of treatment and you signature at the end of the document does not mean that you disclaim from your regular health scheme.

If you experience any adverse events, you could contact the principle investigators by phone any time.

Our expenses for participation in this trial

You do not have to pay for special tests, doctor fee or cost of laboratory tests.

Participation or withdrawn from clinical trial

To participate in this clinical trial is you right to make decision and you could withdraw from the trial at any time. Your decision will not have any risk or consequence to your regular treatment of your diseases.

Our investigator team will withdraw you from the trial for your safety or for other following reasons:

- You could not comply with our protocol.
- You have pregnancy during the trial.

Measurement for protection of participants' data

Your data and your name will be protected from any publicity. In case of publication, the name or address of the participant will be protected and the participant code number will be used instead.

After your agreement, the investigators will have the right to exam your data even after the trial finished. If you could withdraw that right at any time by contacting and inform in person or in writing and send it to the principle investigator (address shown).

If you withdraw from the trial, your add on personal data will not be done. However, some data will be used for evaluation. You could not return to the study protocol again after withdrawal. After your agreement, the investigator could inform your GP regarding the agreement for participation in the trial.

How to manage the residual biologic material

After the study ends, your residual biologic material; blood, will be collected at Division of gastroenterology, Chulalongkorn hospital, Rama IV road, Pathumwan, Bangkok 10330 for evaluate various of biologic markers in the future. We will collect your blood for 15 years and then the remaining specimen will be destroyed after that. If we would like to take any of your collected specimen, we will ask for a permission to Ethic Committee (EC) before using the biologic specimen.

Right of the participants

As you decide to be in this trial, you have the right as following

- You will receive information of the trial
- The investigators will inform regarding method of the study, drugs and other tests. o You will receive information of risk or adverse event from the medications o You will receive information of the benefit of the trial
- You will receive information regarding other alternative treatment that might benefit to your disease.
- You will receive information of management of adverse events or complications.
- You could ask for more information regarding process of the study.
- You will receive information regarding how and when to withdraw from the study which could be any time.
- You will receive the consent form with the signatures and date
- You have the right to make decision whether or not to participate the trial without any influence or pressure from anyone.

If you do receive any compensation for your adverse events related to trial medication or you do not receive proper management as the explanation in this document, you could contact the principle investigators directly or report to Institutional Review Board, Faculty of Medicine,

Chulalongkorn University at the office on the 3rd floor Mahidol Building, King Chulalongkorn Memorial Hospital, Rama 4 Road, Pathumwan, Bangkok 10330, Tel. 02-256- 4455, ext. 14 or 15 during office hour.

Thank you for your cooperation

Consent form for agreement to participate in the trial

Title of research: Comparison of transient elastography, acoustic radiation force impulse and magnetic resonance imaging for non-invasive staging of liver fibrosis in patients with chronic hepatitis C and nonalcoholic fatty liver disease: 1-year follow-up of fibrosis progression

Date of agreement: Date............. Month..................Year.................

I, Mr./Mrs./MS.. Age.............years
Current address..
....Tel. ....
have read the information for the participant and agree to participate in this clinical trial.

I have received the copy of the consent form for participation in the trial and sign with the name and date include receiving the detail document for participant. I have received explanation regarding objective, period of study, methodology, risks that might happen and benefit of this trial. I have enough time to read and ask for any concern regarding the clinical trial and the investigators give all information without any hidden agenda.

I have the right to withdraw from the clinical trial at any time and without the need to explain the reasons. In addition, the withdrawal will not have any consequence to my disease management or my right to receive proper management.

The investigators confirm to protect the secrecy of my data and will reveal only on my permission.

Any investigation or examination of the data by other party including Institutional Review Board member have the right only to examine for the accuracy of the data. By this agreement, I accept for the examination of my previous health history.

The investigator agrees to the participant that, in case of withdrawal, no more additional data will be kept and the data related to the participant will be abolished and could not be traced back to the participant.

I understand that I have a right to exam or correct my personal data and have a right of others to use my personal data by informing the investigators.

I understand that the research data including the health history will not be opened or report by participant name. And the data will be used to process by data correction, computer analysis of the data and then report only for scientific and clinical purpose.

I accept to sign this consent form for participation in the clinical trial with approval.

.... Participant signature

.... Block letters

Date................Month.....................year....................

I have explained the information regarding this clinical trial including objective, methodology, risk or benefit of the trial and the participant as the above name has signed for the agreement to comply with the trial.

.... Investigator signature

.... Block letters

Date................Month.....................year....................

.... Witness signature

.... Block letters

Date................Month.....................year....................
